# Devices used for photobiomodulation of the brain—a comprehensive and systematic review

**DOI:** 10.1186/s12984-024-01351-8

**Published:** 2024-04-10

**Authors:** Filipa Fernandes, Sofia Oliveira, Francisca Monteiro, Michael Gasik, Filipe S. Silva, Nuno Sousa, Óscar Carvalho, Susana O. Catarino

**Affiliations:** 1 Center for Micro-ElectroMechanical Systems (CMEMS-UMINHO), University of Minho, Guimarães, Portugal; 2 Department of Chemical and Metallurgical Engineering, School of Chemical Engineering, Aalto University Foundation, Espoo, Finland; 3 LABBELS—Associate Laboratory, Braga, Guimarães, Portugal; 4 Life and Health Sciences Research Institute (ICVS), University of Minho, 4710-057 Braga, Portugal; 5 ICVS/3BS, PT Government Associate Laboratory, 4710-057 Braga, Portugal; 6 2CA-Braga, CVS/3BS, PT Government Associate Laboratory, 4710-057 Braga, Portugal

**Keywords:** Brain stimulation, Light therapy, Optical device, Photobiomodulation, Neurological pathologies

## Abstract

A systematic review was conducted to determine the trends in devices and parameters used for brain photobiomodulation (PBM). The revised studies included clinical and cadaveric approaches, in which light stimuli were applied to the head and/or neck. PubMed, Scopus, Web of Science and Google Scholar databases were used for the systematic search. A total of 2133 records were screened, from which 97 were included in this review. The parameters that were extracted and analysed in each article were the device design, actuation area, actuation site, wavelength, mode of operation, power density, energy density, power output, energy per session and treatment time. To organize device information, 11 categories of devices were defined, according to their characteristics. The most used category of devices was laser handpieces, which relate to 21% of all devices, while 28% of the devices were not described. Studies for cognitive function and physiological characterisation are the most well defined ones and with more tangible results. There is a lack of consistency when reporting PBM studies, with several articles under defining the stimulation protocol, and a wide variety of parameters used for the same health conditions (e.g., Alzheimer’s or Parkinson’s disease) resulting in positive outcomes. Standardization for the report of these studies is warranted, as well as sham-controlled comparative studies to determine which parameters have the greatest effect on PBM treatments for different neurological conditions.

**Supplementary Information:**

The online version contains supplementary material available at 10.1186/s12984-024-01351-8.

## Background

Photobiomodulation (PBM) is a non-invasive therapy that entails the use of red to infrared (IR) light (wavelengths of 600 to 1100 nm) to stimulate cellular processes that promote tissue healing and regeneration [1–3]. Additionally to being studied and used as a therapy for pain relief [4, 5], wound healing and skin rejuvenation [6, 7], the neuromodulation effects of PBM, when applied to the scalp, have been increasingly more studied, with several clinical studies showing that it could be a safe, non-invasive, and non-destructive alternative to conventional treatments for various neurological disorders.

Functional near-infrared spectroscopy (NIRS) studies have shown that PBM can effectively increase cerebral oxygenation, which has a great impact on cognitive tasks, such as memory and attention, among others [8–13]. For traumatic brain injury (TBI), there are several case studies in which patients exhibited improvement in symptoms, measured through standard neurological tests and self-assessments [14–18]. PBM has also reduced depression symptoms in patients diagnosed with depression disorder [19–22]. For neurodegenerative conditions, such as dementia, Alzheimer’s disease (AD), and Parkinson’s disease (PD), several studies showed improvements in cognition, quality of life, and clinical signs of these conditions [23–26]. Specifically, a randomized controlled trial on the effects of transcranial PBM in patients diagnosed with PD showed improvements in gait, further establishing the relevance of this therapy for neurodegenerative conditions [27].

The effect of PBM on the brain has been studied to understand the mechanisms behind these positive results and to determine which parameters are more beneficial in these treatments. Although brain PBM has been studied for more than two decades, there is great variability in studies using distinct PBM parameters for the same neurological pathologies, such as wavelength, mode of operation (i.e., continuous or pulsed), area of actuation and energy delivered to the head. Often, authors point out the need for further research to confirm methods to establish PBM as an effective treatment for neurological conditions [17, 22, 28–30].

This review aims to draw conclusions from the devices and parameters used for PBM, to determine, if possible, optimal procedures for different pathologies, to promote and accelerate scientific research in this area. Furthermore, since there is some inconsistency in the reporting of these studies, it is also intended to provide further insights into which parameters are more relevant for a full characterisation of the brain PBM.

## Methods

The search of the present literature review followed the guidelines of the Preferred Reporting Items for Systematic Reviews and Meta-Analyses (PRISMA) method [31].

### Data sources, search strategy, and eligibility criteria

The PubMed, Scopus and Web of Science databases were used to perform a comprehensive electronic search for articles in which a device was used to stimulate the human brain by means of light in the red to IR wavelengths. The search strategy was conducted in each database up to May 23rd 2022, and it is described in Additional file 1: Table S1. The records yielded by the search were exported to the Microsoft® Excel software. Duplicate results were identified with a software feature and, after a manual verification, these were removed. The title, abstract and keywords of the remaining records were independently reviewed by author FF, and studies that did not relate to the brain, did not include light stimulation, and concerned surgical procedures (e.g., thermal laser ablation) were excluded.

On September 23rd 2022, the Google Scholar database was used to scan for records that may have been left out of the previous search. The first combination of keywords used was “transcranial + PBM”, and the second combination was “LLLT + brain”. Lastly, the citations of relevant reviews were scanned for further analysis.

The remaining records were identified according to the following inclusion and exclusion criteria. The process was carried out by the author FF and later corroborated by the authors SO, FM, OC, SC.

- Inclusion criteria: using a light device in the red to IR wavelengths; the device should be applied in the head or neck area; the purpose of the stimulus should be the stimulation of the brain; wavelength should be reported.
- Exclusion criteria: reviews, meta-analysis, conference papers, books, studies concerning statistical analysis of previous clinical trials, letters to editors, study protocols; studies not available in English; near-infrared spectroscopy for cerebral oxygenation or monitoring; in vitro and animal studies; studies that did not include a device which could be used for brain stimulation; simulations.

### Data collection

From the included articles, relevant data was retrieved, namely the device name/company, device composition/type of light source, pathology/condition studied, subjects, area of actuation, location of the stimulus, wavelength, mode of operation, power density or irradiance, energy density or fluence, power output, energy per session, session time, outcomes measured and results. The data collection was performed by the author FF. In some instances, if the article referenced a previous study in which the parameters used were similar, it was further consulted to fill in the missing information. Otherwise, when information was missing, the parameter was marked as not reported (NR). Different articles described the use of the same device, but with different operating parameters and, therefore, no information was assumed. The final table obtained was reviewed by all authors.

This information was then organized in different ways, firstly to compare the devices used, secondly to understand trends in paraments, and finally to determine pathological details.

## Results

### Search and selection of studies

**Fig. 1:**
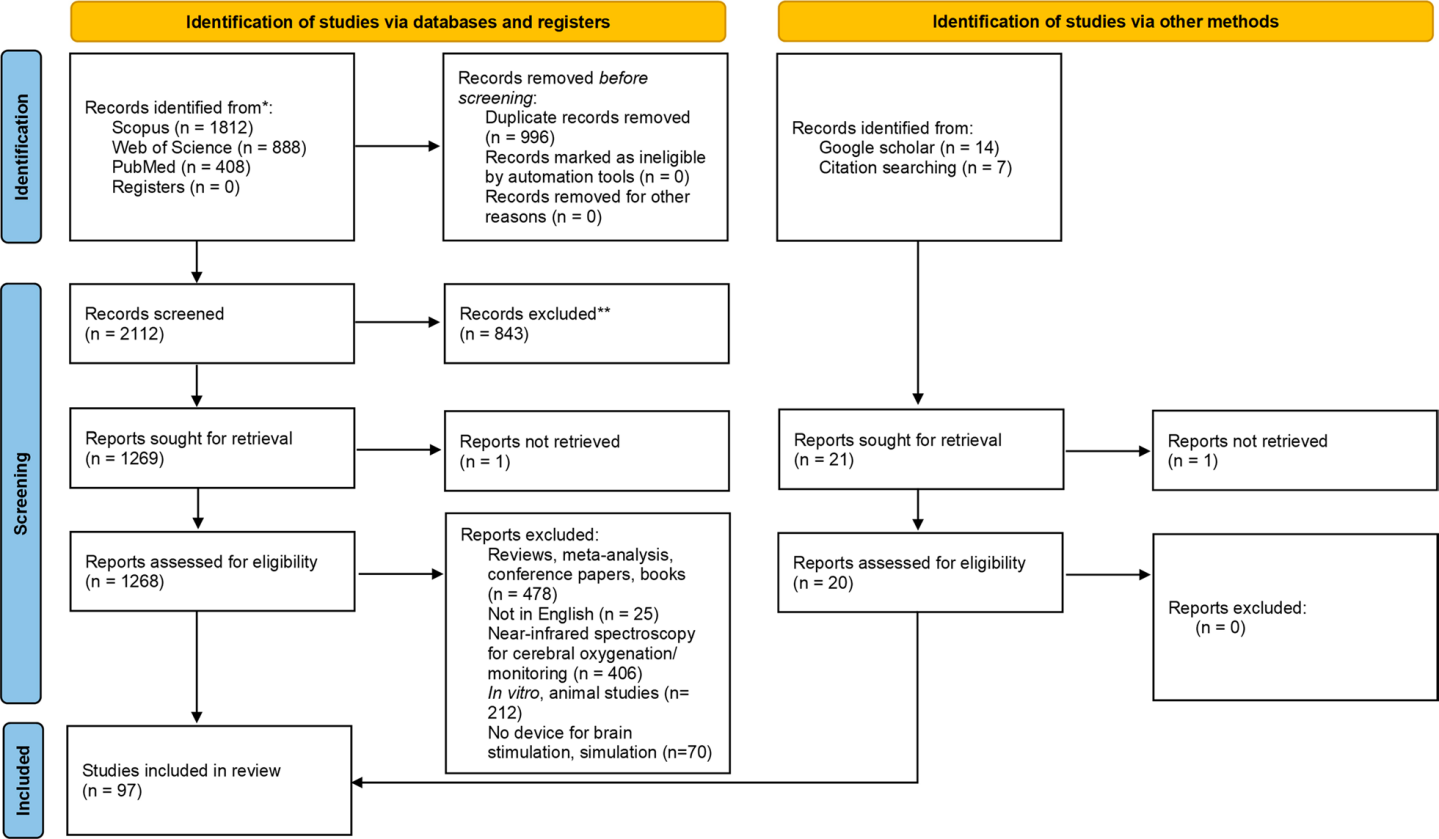
Preferred reporting items for systematic review and meta-analysis (PRISMA) flowchart of included and excluded studies

A total of 3108 records were retrieved from the Scopus, Web of Science, and PubMed databases. Afterward, 996 duplicate records were removed, and 2112 articles were screened, from which 843 records were excluded for not being relevant to the review at hand. A total of 1269 records were screened, from which one could not be retrieved after contacting the corresponding author. Seventy-seven articles were ultimately selected according to the eligibility criteria. From the Google Scholar database, 14 articles were selected, among which one could not be retrieved. Finally, 7 articles were selected among the citations of records included in the search. Thus, a total of 97 articles were included in this review. Figure 1 depicts the search and selection process yielded in the search.

### Devices

Of the 97 articles reviewed, 92 relate to brain PBM in human subjects. Most articles used transcranial devices (n = 90), from which 12 simultaneously used intranasal stimulation, two the eyes, and one the ears. Two articles used a solo intranasal device [32, 33]. Since some articles reported the use of more than one device, either for comparison, simultaneous use, or other reasons, there are 104 reports of devices used for PBM of the brain. One of the reported devices, which was provided to the patients for home use (MIDCARE laser device), was not considered since it was similar to the one used in the clinic (Irradia MID 2.5 laser device), and for that reason, did not bring new information to this review [23].

Besides the articles related to brain PBM, five articles and five reports describe light penetration studies. From those, three studies used human skulls, three used cadaver heads and one used both of them. Only one study applied an intranasal device in a cadaveric head, and the remainder studies utilized transcranial devices [34–38].

A total of 55 different devices were used for the reported brain PBM and light penetration studies, across 109 reports, implying that some reports refer to the use of the same device. In most studies, the parameters used for each device were similar, with few exceptions. The devices were grouped into 11 categories, according to their design or lack of a description. A brief description of each category is provided below, as well as the distribution of the different types of devices over the reports.

#### Laser handpiece

The most common type of device was the laser handpieces, concerning 14 devices and 39% of all reports (n = 109). This category of devices consists in a handpiece that is manually handled by an operator and can be placed anywhere on the head. These devices are generally of simple construction, requiring only a control unit and a fibre optic cable that connects to the handpiece.

#### LED clusters

The second most common system are light-emitting diode (LED) clusters, with six devices being used in 17% of the reports. LED clusters construction may be similar to that of laser handpieces, comprising a control unit that connects to the LED cluster piece. However, the number of LED diodes is generally higher, fibre optics are not used, and the structure that contains the diodes is flatter. On the other hand, some LED clusters are comprised of a single component that contains the LEDs and control unit, and only allow the user to turn it on and off.

#### LED covered helmets

LED covered helmets were also common, relating to 12 devices, and 12% of the reports. Regarding the design of helmet devices, they can be comprised of LED clusters that are arranged in a helmet shape; LEDs incrusted in a metal or plastic structure, shaped like a helmet; or neoprene pads filled with LED rows, that are place around and on the top of the head.

#### LED and laser devices without description

Reports where the commercial title of the device was not indicated, a design was not described, or an image was not provided, were grouped in the categories “LED devices without detailed description” or “Laser devices without detailed description” since the type of light source used was the only clear distinction. LED and laser devices without detailed description refer to a total of eight devices and 7% of reports, and four devices and 4% of reports, respectively.

#### Localized LED helmets

For localized LED helmets, there were reports for two different devices used in 11% of the reports. These devices resemble a helmet but are comprised of two metallic straps placed on the top and crown of the head, onto which a reduced number of LEDs are attached in specific locations, namely three posterior transcranial LEDS, one anterior transcranial LED, and one intranasal LED.

#### Intranasal LED

Intranasal LED devices relate to three types of devices and were used in 5% of reports. This type of device consists of a single LED that is placed inside the nostril.

#### Intranasal laser

There were two intranasal laser devices, which account for 2% of all reports. This type of device is comprised of a single laser that is placed inside the nostril.

#### LED headband

The LED headband category relates to two devices and 2% of all reports. This type of device does not fit in the abovementioned categories, as it only stimulates one area of the head, namely the forehead. One of the devices is not described in detail, but the other is commercially available for NIRS and contains a headband that is placed on the forehead to measure oxygenation, being composed of sensors and LEDs, and a plastic cover to disguise the mechanical components. This apparatus is connected to a control unit that can be portable.

#### Laser-covered helmet

There is one laser-covered helmet, relating to 1% of all reports. This device comprised LEDs incrusted in a plastic structure, shaped like a helmet.

#### Laser needle

The laser needles category refers to a single device, representing 1% of all reports. This device comprises four stainless steel laser acupuncture diode needles, which are connected to a control unit by fibre optics. The four needles are held in place with wire holders attached to a crown that wraps around the head of the participant.

**Fig. 2:**
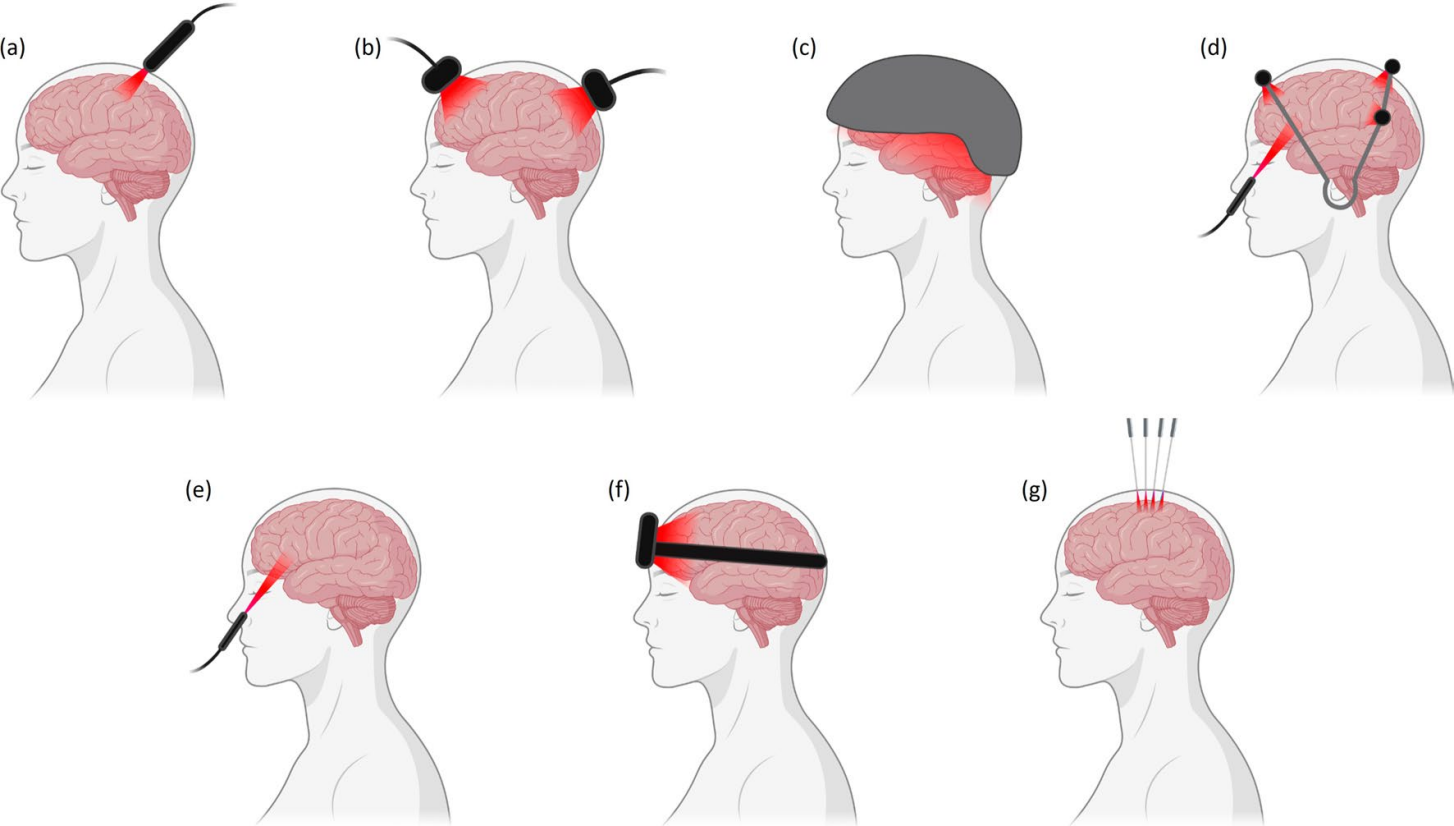
Light stimulation device category illustration (not in scale): **a** laser handpiece; **b** LED cluster; **c** LED/laser helmet; **d** localized LED helmet; **e** intranasal LED/laser; **f** LED headband; **g** laser needles. Created using BioRender.com

Figure 2 presents a general representation of the devices previously mentioned, namely laser handpieces (Fig. 2a), LED clusters (Fig. 2b), LED or laser helmets (Fig. 2c), localized LED helmets (Fig. 2d), intranasal LED or lasers (Fig. 2e), LED headbands (Fig. 2f), and laser needles (Fig. 2g), along with a schematic representation of their positioning.

LED and laser devices without description account for 22% (n = 55) of all devices, and 11% of all reports. Overall, LEDs are more frequently used than lasers in these devices, relating to 60% of all devices, and 54% of all reports.

Table 1 reports the assortment of devices used across the 97 articles. For each device, it is referred the number of reports in which it was used. In particular, three of the articles used LED helmets with a description of the device but without a name and, thus, the devices were grouped and designated as “LED helmets without name information”. The same rationale was applied to two intranasal devices and the laser helmet without name information included in the article. Furthermore, the purpose of the study (pathology or light penetration) is indicated for each device. Purposes highlighted in bold refer to the most common application for a specific device (comprising, at least, half of its reports). Multiple inputs concern articles that used different parameters for the same device, or the categories which group the devices without description. Additionally, the use of simultaneous wavelengths is identified, with the exception of a study which used sequential red and NIR wavelengths [33].

**Table 1:** Categories and parameters of the reviewed devices

Device (company)	No. of reports	Category	Description	Location	Actuation area (cm^2^)	Wavelength (nm)^a^	Mode of operation	Power density (mW/cm^2^)	Energy density (J/cm^2^)	Energy per session (J)	Power output (W)	Study
Bloodcare Medical Laser Device (Shanghai Taicheng Technology&Development Co. Ltd., CN) [32]	1	Intranasal laser	GaInP/AlGaInP diode	Intranasal	0.358	650	NR	8.38	NR	NR	NR	Blood conditions
Intranasal laser without name information [37]	1	Intranasal laser	Optical fibre-based frontal light diffuser	Intranasal	0.0057	671 or 808	NR	NR	NR	NR	0.8; 1	Light penetration
Vielight Intranasal 810 (Vielight Inc., CA) [14, 25, 26]	3	Intranasal LED	1 LED diode	Ventromedial prefrontal, entorhinal cortex, hippocampus	1	810	10 Hz 50% DC	14.20	10.65	10.65	0.014	AD; Dementia; Gulf War Illness
Intranasal LED without name information [33]	1	Intranasal LED	NR	Brainstem	NR	660	NR	NR	NR	NR	NR	PD
Vielight Intranasal 633 (Vielight Inc., CA) [14]	1	Intranasal LED	1 LED diode	Ventromedial prefrontal, entorhinal cortex, hippocampus	1	633	Continuous	8	12	12	0.0080	Gulf War illness
Laser without detailed description [21, 38–40]	4	Laser	NR	PFC bilaterally; left motor cortex; several regions;	0.0314–35.8	808, 820, 830, 852 or 1064	Continuous; 10 Hz and 100 Hz 20% DC	54.8; 167; 310	Mostly NR; 65.8	1635; 2300	2; 2.3; 5	Physiological characterisation; cognitive function; depression; light penetration
Model CG-5000 Laser (Cell Gen Therapeutics LLC, US) [9, 11, 12, 20, 29, 41–54]	19	Laser handpiece	Laser with collimated beam, connected to fibre optic cable	Right PFC; dorsolateral PFC bilaterally	12.56–13.85 per site	1064	Continuous	162–257.40	60–137.5	1496–1680	2.20–3.50	**Cognitive function; physiological characterisation**; depression; fear
NeuroThera® Laser System (Photothera, Inc., US) [22, 55–57]	4	Laser handpiece	Class IV laser	Frontal, parietal, temporal, and occipital regions; PFC bilaterally	7.1 per site	808	Mostly NR; continuous	Mostly NR, 700	NR, 1 or 84	Mostly NR; 2400	Mostly NR; 5	**Stroke**; depression
CytonPro (CytonSys Inc., US) [10, 58]	2	Laser handpiece	Collimated laser	Left and right PFC	13.6 per site	1064	Continuous	250	120 or 150	1632^b^	3.40	Bipolar; physiological characterisation
Diowave 810 (Diowave, US) [59, 60]	2	Laser handpiece	Class IV laser probe	PFC and temporal regions bilaterally	NR	810	Continuous and 10 Hz	NR	55 to 81	NR	10–15	TBI; TBI with depression
Model K-1200 dual-wavelength (K-Laser, US) [34, 36]	2	Laser handpiece	Laser with collimated beam, connected to fibre optic cable	Calvaria, bregma	1.075 per site	800 and 970 or 800	NR	200–700	NR	NR	NR	Light penetration
Power Twin 21 (MKW Lasersystem GmbH, DE) [61, 62]	2	Laser handpiece	21 laser diodes	Superior edge of fossa sphenoidale, upper edge of the two fossa sphenoidalis	NR	785	Continuous	10	1	126	NR	Severe disorder of consciousness; TBI
Theralase® TLC-2000 CLT (Theralase Technologies Inc., CA) [63, 64]	2	Laser handpiece	5 near-infrared (NIR) laser diodes and 4 RED laser diodes	Midline occipital region, Circle of Willis, and over the mastoid processes bilaterally; PFC various locations	NR	660 and 905 or 905	Superpulsed (200 ns)	NR	NR	315	0.05 or 0.13	ADHD; TBI
Irradia MID 2.5 laser (Irradia, SE) [23, 28]	2	Laser handpiece	4 laser diodes	back of neck; several regions, basal ganglia and susbtantia nigra (intraoral)	0.196 or 0.635	904	50 Hz	47	NR	10 or 39.6	0.12 or 0.3	PD
Ultralasers MDL-N-808-10000 (Ultralasers, CA) [65, 66]	2	Laser handpiece	1 diode laser connected through an optic fibre to a laser driver	Right PFC	0.785	808	NR	318	NR	150	0.25	Physiological characterisation
Aspen Pinnacle diode (Aspen Laser LLC, US) [67]	1	Laser handpiece	Commercial model	Temporal lobe, PFC, left hippocampus	7.065 per site	810	10 Hz	177–354	5–60	110–4000 per location	5–25	AD
CTL-1100 low power laser (Toptica, DE) [68]	1	Laser handpiece	Commercial model	Cervical	1 per site	810–830	6 kHz	NR	4	NR	0.10	Vertebrobasilar insufficiency
Endolaser 476 (Enraf Nonius, NL) [69]	1	Laser handpiece	GaAs laser probe	Primary motor cortex	1 per site	905	3 kHz	50	15	NR	NR	Physiological characterisation
Laser Lumix 3 Plus Ultra model (Fisioline S.r.l., IT) [70]	1	Laser handpiece	Commercial model	Primary motor cortex bilaterally	3.14 per site	808	Continuous	200	60	NR	NR	Motor performance
LiteCure® LT1000 (LiteCure LLC, US) [59]	1	Laser handpiece	Class IV laser probe	PFC and temporal regions bilaterally	NR	810 and 980	Continuous and 10 Hz	NR	55 to 81	NR	10–13	TBI with depression
Laser helmet without name information [71]	1	Laser helmet	12 RED and 12 IR LEDs arranged on a helmet	Whole head	NR	630 and 810	75 Hz 20% DC	800	144	NR	2.4	Schizophrenia
Laser WeberMedical Klasse 1, Type BF, Laser Class 3B (Weber medical GmbH, DE) [72]	1	Laser needles	Collimated laser beam connected to fibre optic cable and four stainless steel laser diode needles	Primary motor cortex	0.35	810	Continuous	500	NR	NR	0.15	Physiological characterisation
LED without detailed description [73–80]	8	LED	NR	PFC; dorsolateral PFC bilaterally; midbrain (back of neck); whole head without PFC; frontal sinus; frontal and parietal lobes	1.32–80, mostly NR	627, 810, 940 or 945	Mostly NR; continuous; 5–20 Hz 50% DC	6–250	9.35–65.7	NR	Mostly NR, 0.11, 0.20	**Drug abuse**; anxiety and depression; physiological characterisation; PD
MedX Health Model 1100 (MedX Health Services Inc., CA) [14, 17, 18, 30, 81]	6	LED cluster	One, two or three LED clusters. 49 or 61 diodes (9 RED + 40 or 52 NIR diodes)	PFC various locations; frontal, occipital, parietal, and temporal regions; ears	19.39 or 22.48 per cluster	633 and 870 or 870	Continuous or 146 Hz	4–44.40	1–39	42.30–1349	0.09, 0.5 or 1 per cluster	**TBI**; cognitive function; Gulf War illness; stroke
Omnilux New U device (Photomedex Inc., US) [19, 35, 82, 83]	4	LED cluster	Two similar clusters used simultaneously, except for light penetration	Dorsolateral PFC bilaterally; frontal, left, and right parietal skull and frontal, temporal, and occipital skull	28.7 per cluster	633, 823, 830 or 633 and 830	Continuous	33.2–72.6	40–65.2	Mostly NR; 3440	Mostly NR; 1 per cluster	Autism; depression; sexual dysfunction; light penetration
InGaAIP LED (Iranbargh, IR) [84–86]	3	LED cluster	20 diodes—square	Right PFC	1.4	850	Continuous	285	42.75 or 60	60^c^	0.4	Cognitive function; physiological characterisation
Wisefor i5-3800 (Wisefor LTD, HK) [8, 87, 88]	3	LED cluster	9 or 5 diodes	PFC various locations	5^d^	810	Continuous	20	7	NR	NR	Cognitive function; dementia
LiteCure® TPBM-1000 (LiteCure LLC, US) [89, 90]	2	LED cluster	4 LED clusters	PFC bilaterally; dorsolateral PFC bilaterally	35.8	830	Continuous (c) or pulsed (p) 10 Hz 33% DC	54.80	c—65.80 or p—21.70	c—2300 or p—800	c—2 or p—0.70	AD; physiological characterisation
L-light (SUN-MECHATRONICS, JP) [91]	1	LED cluster	23 diodes—square	PFC bilaterally	21.85 per cluster	850	Continuous	11.40	20.5	538	0.299	Physiological characterisation
Cerebral Science (Center for Anxiety and Traumatic Stress Disorders, US) [92]	1	LED headband	NR	PFC various locations	80	830	Continuous	30	36	2900	2.4	Anxiety
OEG-SpO2 (Spectratech Inc., JP) [93]	1	LED headband	Six light emitters and six light detectors	Central PFC	NR	770 and 840	12.21 Hz	25	NR	NR	NR	Cognitive function/function
LED helmet without name information [16, 24, 94, 95]	4	LED helmet	18/12/13 clusters of 20/70 or 14/4 LEDs	Whole head (two with ocular stimulation)	400; ~ 650	630, 810 or 1060–1080	Mostly NR; continuous	23.1; 36	3.74; 43	Mostly NR; 1494	10.79; 15	AD; dementia; TBI
Cognitolite Transcranial Photomodulation System (Cognitolite LLC, US) [96, 97]	2	LED helmet	14 or 15 arrays of 70 LEDS—air cooled	Whole head (with eyes)	NR	1060–1080	10 Hz 50% DC	12 per module	NR	1368	3.80	Cognitive function/function; AD
Duo Coronet (Wellred, AU) [33]	1	LED helmet	Aluminium sheets lined with LED strips. 670 and 810-nm (n = 150), 850-nm (n = 120)	Whole head	NR	670, 810 and 850	Continuous	NR	NR	NR	39.36^e^	PD
InLight Wellness Systems (Inlight Therapy Inc., US) [15]	1	LED helmet	2 neoprene pads with 180 RED and 222 NIR LEDs. One pad circled the skull, the other covered the top of the head	Whole head	519	629 and 850	73, 587, and 1175 Hz 35% DC	6.40	7.7	3994	3.3	TBI
LumiWave (BioCare Systems, Inc., US) [98]	1	LED helmet	784 NIR GaAIAs LEDs in a cap configuration: four linear pod sets each with 4 LED clusters with 49 LEDs	Occipital, temporal, frontal and parietal regions	360	903	NR	16.67	20	NR	NR	TBI
Photobiomodulation Helmet (Suyzeko, CN) [13]	1	LED helmet	256 diodes	Whole head	NR	810	NR	24	NR	NR	~ 15	Physiological characterisation
PhotoMedex LED lined helmet (Photomedex INC., US) [14]	1	LED helmet	18 LED clusters (4.5 × 4.8 cm)—20 diodes per cluster	Whole head	388.8	830	Continuous	29	26	19,043	~ 11.30	Gulf War illness
Thor photomedicine LED lined helmet (THOR Photomedicine Ltd, UK) [14]	1	LED helmet	5 or 10 LED clusters^f^—69 diodes per cluster. Midline clusters or right and left ear clusters	Whole head, ears	141.5 or 283	660 and 850	Continuous	41 or 35	26	4063 or 8130	6.328 or 10.754	Gulf War illness
Transcranial helmet (ProNeuroLIGHT LLC, US) [25]	1	LED helmet	Helmet—150 NIR LEDs and 50 RED LEDs	Whole head	NIR—18, RED—6	635 and 810	Continuous	NIR—31, RED—75	NIR—46.5, RED—112.5	NIR—837, RED—675	NIR—0.558, RED—0.450	AD
Vielight® Neuro Alfa (Vielight Inc., CA) [26, 99–103]	6	Localized LED helmet (intranasal and transcranial)	3 posterior transcranial, 1 anterior transcranial, and 1 intranasal LED. Aluminium strips frame	Ventromedial prefrontal, entorhinal cortex, hippocampus (intranasal), central PFC, top of frontal lobe, temporal lobe bilaterally	1 per LED	810	10 Hz or 10 Hz 50% DC	Posterior—100, anterior—75, intranasal—25; transcranial—41, intranasal—23	Posterior—60, anterior—45, intranasal—15; transcranial—24.6, intranasal—13.8	240; 309	Posterior—0.10, anterior—0.075, intranasal—0.025; transcranial—0.041, intranasal—0.023	Autism; cognitive function; dementia; Gulf War illness; physiological characterisation; TBI
Vielight® Neuro Gamma (Vielight Inc., CA) [23, 99, 103–106]	6	Localized LED helmet (Intranasal and transcranial)	3 posterior transcranial, 1 anterior transcranial, and 1 intranasal LED. Aluminium strips frame	Ventromedial prefrontal, entorhinal cortex, hippocampus (intranasal), central PFC, top of frontal lobe, temporal lobe bilaterally	1 per LED	810	40 Hz or 40 Hz 50% DC	Posterior—100, anterior—75, intranasal—25	Posterior—60, anterior—45, intranasal—15	180; 240	Posterior—0.10, anterior—0.075, intranasal—0.025	AD; autism; cognition/function; PD; TBI

*NR* non reported, *AD* Alzheimer’s disease, *ADHD* attention deficit hyperactivity disorder, *DC* duty cycle, *NIR* near-infrared, *PD* Parkinson’s disease, *PFC* prefrontal cortex, *TBI* traumatic brain injury

^a^For wavelength, “or” means the wavelengths were used individually, “and” means they were used simultaneously

^b^For 120 J/cm^2^

^c^For 42.75 J/cm^2^

^d^For 5 diodes

^e^Efficiency unknown

^f^Respective values

### Location of the stimulation

There were various methods for reporting the location of stimulation, with some articles using the 10–20 electroencephalogram (EEG) electrode system, others the Brodmann areas, while some studies did not use any standard methods, but rather a description of the location. To uniformize the information, instead of the head areas in which the device was placed, the target brain area was determined for each situation (e.g., if the right side of the forehead was being stimulated, it was identified as the right prefrontal cortex (PFC). When the description provided in the article did not indicate the target brain area, the article description was considered [14, 23, 24, 30, 61, 62, 64, 78, 96].

The number of reports relates to each mention of a stimulation location, across all articles, and resulted in a total of 212 reported regions, with only one article not mentioning the location of the stimulus [60]. The PFC was the most stimulated region of the brain, with 87 reports of stimulation in this area, either exclusive or simultaneous. The reports were usually of bilateral stimulation or only on the right side of the PFC. Additionally, the frontal region was reported 19 times, and the motor cortex four times, being the frontal lobe the most stimulated region of the brain, with 52% of all reports. The frontal lobe was followed by the temporal region, which was mentioned 18 times. Additionally, the entorhinal cortex was referred 16 times, and the Wernicke’s area was mentioned once. These two areas also concern the temporal lobe, which results in a total of 17% of reports relating to the temporal lobe.

A trend for the stimulation of several regions during the same treatment was noted, concerning 43 of the initial 104 reports for PBM of the brain. Henceforth, the term several regions will be used to describe situations where three or more regions of the brain were stimulated during treatment. This threshold was defined since two regions usually relate to stimulation of the forehead in two different locations.

### Stimulation parameters

The functioning parameters considered for the devices’ operation were wavelength, mode of operation, power density, energy density, energy per session, power output, area of actuation and session time. Of the 109 reports, 34% reported all parameters, and the remaining studies had at least one parameter missing. Since wavelength was one of the inclusion criteria, all articles provided this parameter. Energy per session was the parameter with the least mentions, missing in 52% of reports, followed by energy density, missing in 33%, power output, missing in 30%, area of actuation, which was missing in 26%, and mode of operation, missing in 25%. Power density and session time were usually provided, missing only in 16% and 11%, respectively.

Some reports provided more than one value for each parameter, either because different parameters were used simultaneously or because different circumstances were tested. For power output and area of actuation, when the value provided was related to only one light cluster, but several were used simultaneously, the value was multiplied by the number of clusters.

**Fig. 3:**
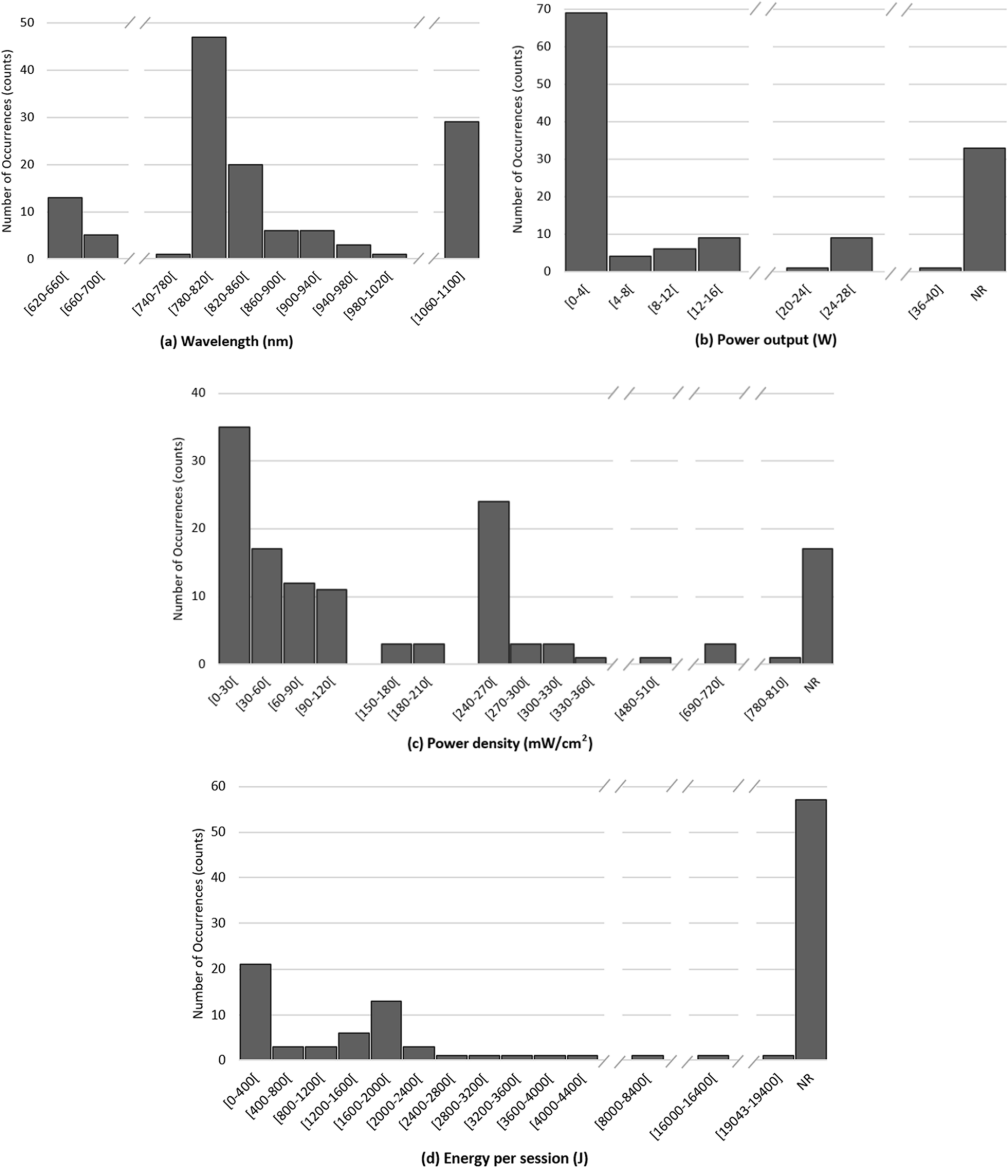

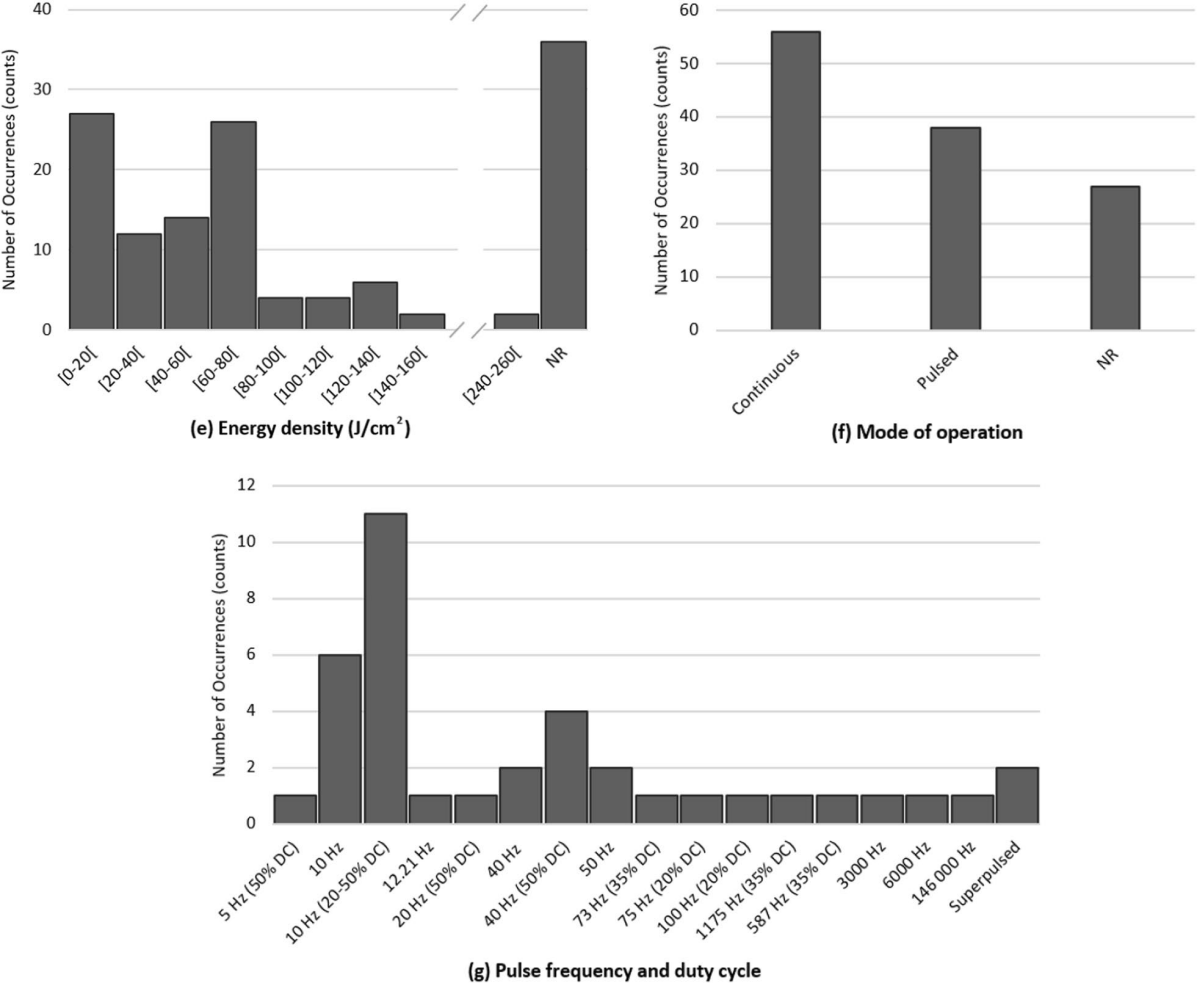
Frequency of occurrence of each parameter value: **a** wavelength, **b** power output, **c** power density, **d** energy per session, **e** energy density, **f** mode of operation, **g** pulse frequency and duty cycle. *NR* non reported

Considering the reported values for each parameter, the most common value for wavelength was 810 nm (26%, n = 131), most devices operate in continuous mode (60%, n = 94), the most frequent power density was 250 mW/cm^2^ (20%, n = 117), the most common energy density was 60 J/cm^2^ (22%, n = 97), the most prevalent energy per session was 1632 J (16%, n = 57), with most of the devices working with a power output of 3.4 W (15%, n = 99), and usually actuating over an area of 13.6 cm^2^ and 5 cm^2^, both with identical scores (12% each, n = 81). Finally, the most frequent session time was 20 min (22%, n = 107). Figure 3 shows the frequency of occurrence of the wavelength (Fig. 3a), power output (Fig. 3b), power density (Fig. 3c), energy per session (Fig. 3d), energy density (Fig. 3e), mode of operation (Fig. 3f), and a detailed view of pulsed modes (Fig. 3g), to better demonstrate tendencies and parameter distribution.

### Health conditions

For the analysis of the health conditions, light penetration studies were not considered. A total of 23 different health conditions were identified across 92 articles. To avoid the repetition of information, articles that studied two combined conditions, namely anxiety with depression, and traumatic brain injury with depression, were considered together as a different condition. Physiological characterisation was the most commonly studied condition, referring to 21 articles including studies of cerebral oxygenation, hemodynamic, and cortical excitability. Cognitive function was assessed in 18 articles, which evaluated cognitive performance after or during PBM. TBI was studied in 10 articles, and Alzheimer’s disease was addressed in seven reports. With four articles each, depression, Parkinson’s disease, and stroke were also addressed. Other health conditions, as dementia and drug abuse were studied in three articles each. Anxiety and depression, autism, fear, and PTSD (Gulf War illness) relate to two studies each. With one article in each instance, there were also studies for attention deficit hyperactivity disorder (ADHD), anxiety, bipolar disorder, motor performance, schizophrenia, severe disorder of consciousness, sexual dysfunction, TBI with depression, blood conditions, vertebrobasilar insufficiency (VBI). Of the 57 sham-controlled studies, 53% refer to physiological characterisation and cognitive function studies.

Considering that one article did not specify the number of subjects participating in the trial [13], the remaining studies totalized 3804 participants, with 70% of them being diseased patients and the remaining healthy volunteers. More than half the subjects (56%) were related to cognitive function or stroke studies. The sex of the subjects was not reported in 13 articles (30%) [2, 3, 5, 31, 35, 38, 48, 58, 61, 64, 66, 88, 91], while, in studies reporting this information, 51% of all subjects were female. The subjects were aged between 18 and 90 years, except in four studies in which subjects under 18 years (i.e., 5 to 17 years) were used [63, 78, 98, 99].

Neuropsychological assessment tests, such as the California Verbal Learning Test II (CVLTII) for TBI, the Hamilton Depression Rating Scale (HDRS) for depression, and the National Institutes of Health Stroke Scale (NIHSS) for stroke, were the most common outcome measured, which were used in 17 types of health conditions. Self-assessments were employed in seven health conditions, and medical brain imaging techniques, such as electroencephalogram (EEG), magnetic resonance imaging (MRI), functional magnetic resonance imaging (fMRI), NIRS, and motor evoked potentials (MEPs) induced by transcranial magnetic stimulation (TMS), were used in six health conditions. Table 2 summarises the abovementioned data.

**Table 2:** Health conditions, subjects, outcomes measured, and main reported results

Condition	No. of articles	No. of subjects	Condition of subjects	Age	Sex	Sham controlled	Treatment time	Outcomes measured	Results
Physiological characterisation [9–13, 39, 42, 43, 47, 52, 53, 65, 66, 69, 72, 76, 79, 86, 90, 91, 102]	21	553	552 healthy volunteers. 1 patient	18–85 years	256 females. 79 not specified (3 articles)	14	2, 2.5, 4, 5, 8, 10, 11, 15, 20, 30 min sessions	**Brain imaging**—EEG, NIRS, fMRI, transcranial doppler, DCS. Motor cortex excitability—TMS-evoked MEPs. Neuropsychological assessment tests—cognitive task (2-back)	Increased electrophysiological oscillations, neuromodulation of alpha and gamma powers, induced neuroplastic changes in the cortex. Increased cortical excitability and cerebral oxygen saturation. One article showed that PBM had no effect on the four major resting-state brain networks, relating to the fact that the subjects were young and healthy, as opposed to other studies. One article showed significant temperature increase
Cognitive function [8, 29, 40, 41, 44, 48–51, 54, 70, 81, 85, 88, 93, 96, 100, 104]	18	697	645 healthy volunteers. 52 patients	Mostly 18–35 years. 5 studies above 49 years	286 females. 138 not specified (4 articles)	16	2.5, 5.8, 6, 7.5, 8, 12, 20 min sessions	**Neuropsychological assessment tests**—ANAM, PGNG, KBIT, attention task, MEFT, CFT, simple RTT, PVT, DMS, WCST, category task, PANAS, HKLLT, REY-O. Brain imaging—**EEG, NIRS**, fMRI	Facilitates behavioural cognitive processing in adults at risk for cognitive decline and age-related memory deficits. Enhances visual working memory capacity, sustained attention, cognition, and emotion. Significant change in brain electrophysiological features. Improved performance and function. Clear influence on brain activity, but only in regions that were functionally active. Effective and safe
Traumatic brain injury [15–18, 60, 61, 64, 95, 98, 103]	10	142	111 patients. 31 healthy volunteers	14–71 years	11 females. 99 not specified (2 articles)	2	10, 20, 25.8 to 64.5, 30, 42 to 60 min sessions	**Brain imaging**—SPECT scans, MRI, EEG, cerebral blood flow. Neuropsychological assessment tests—CVLT-II, WAISIV, TMT-B, DVT, r-CRS. Self-assessments	Self-assessments and test results showed improvement in cognitive function, concentration, reaction time, verbal memory, and overall symptoms. Affects myelin repair pathways and increases synapses after acute and chronic TBI
Alzheimer’s disease [24, 25, 67, 89, 97, 105, 106]	7	92	82 patients. 10 healthy volunteers	20–85 years	43 females	4	6, 20, and 25 min sessions	**Neuropsychological assessment tests**—MMSE, ADAS-Cog, MoCA, WMQ, AST, PSM, IADL, CDT, CPT, LMT-I and II, TMT-A and B. Brain imaging—**EEG**, MRI, DCS, biomarkers. Self-assessments	Positive improvements enhanced cognitive functions and reversed olfactory dysfunction, safe and well tolerated, potentiated fast oscillations. Delta waves power increase improved alertness and attention; alpha waves decrease caused less anxiety
Depression [19–22]	4	125	Patients	19–64 year	80 females	4	8, 20 to 30 min sessions	Neuropsychological assessment tests—**HDRS**, **T-SRQ**, **QIDS-C**, CES-D, dot probe task, ABM & ABM Responsiveness	Reduction of depression symptoms, anti-depressant effects. 1 article established a threshold of inefficacy of t-PBM for MDD
Parkinson’s disease [23, 28, 33, 75]	4	58	Patients	50–80 years	21 females	1	2.8, 5.5, 10 to 15, 20, 30, 35 min sessions	Neuropsychological assessment tests—UPDRS, MoCA, TUG, cognition, fine motor skill and static balance tests. Self-assessments	Improvements in signs of PD in 2 studies. One noted minimal positive changes. One demonstrated that H2 water + PBM alleviated severe disease symptoms
Stroke [30, 55–57]	4	1416	Patients	40–90 years	339 females. 630 not specified (1 article)	3	19.5 to 39, 40 min sessions	Neuropsychological assessment tests—**NIHSS**, BDAE, BNT, PNT, mRS, Letters FAS test. Brain imaging—fMRI	Different light locations caused different behavioural effects, may serve as a new treatment that can promote better neuromodulation poststroke. One study determined it was safe and effective. Two studies stated it had no significant outcomes
(Non-specified) dementia [26, 87, 94]	3	65	Patients	48–90 years	27 females	1	5.8, 6, 20, 25 min sessions	Neuropsychological assessment tests—MMSE, ADAS-Cog, CDR, FAQ, HKLLT, Rey-O, GAS-10, CGDS	Overall improvement in symptoms—cognitive, executive, mood swings, visual and verbal memory, independence. Less depressive and anxiety symptoms. Safe with no side effects reported
Drug abuse [73, 74, 80]	3	103	Patients	18–70 years	12 females. 39 not specified (1 article)	2	4 and 8 min session	Neuropsychological assessment tests—**OCS**, **HDRS**, **HARS**, LVFT, Timeline Follow back, PANAS, Wellbeing/Distress Scale, CTHEV. Drug screens. Reported improvements	Reduces opioid cravings and use, as well as depression and anxiety
Anxiety and depression [77, 78]	2	32	22 healthy volunteers. 10 patients	17–60 years	23 females	2	1.4 and 4 min sessions	Neuropsychological assessment tests—HADS, PANAS, SCID, HDRS, HARS, test faces, drawings, holding force and grip strength. Brain imaging—NIRS	Lower anxiety and depression scores. Comfortable and safe treatment. In one study, one test showed improvement the other did not
Autism [82, 99]	2	31	Patients	5–59 years	9 females	0	20 to 30 min sessions	Neuropsychological assessment tests—SRS-2, CGI-I, CGI-S, ASRS, BRIEF-A, Q-LES-Q, GAF, CARS, HSQ-ASD, APSI, SDAG, MERS-R, PSQI	Reduced symptoms and severity—improvement in executive functions, such as cognitive flexibility, emotional control, sleep quality, attention. Well tolerated and effective
Fear [45, 46]	2	232	Healthy volunteers	18–65 years	96 females. 120 not specified (1 article)	2	8 min session	Self-assessments	No improvement in exposure, but anxiolytic effects may be achieved. Combination of behavioural training with non-invasive brain stimulation may be a treatment
PTSD (Gulf War illness) [14, 101]	2	50	Patients	52.4 mean age	All male	1	4, 10.7, 12.6, 20, 25, 28.2 min sessions	Neuropsychological assessment tests. Self-assessments	Improvement in symptoms, treatment should be continuous to continue the effects. Safe with no side effects. Treatments will likely need to be continued on a regular basis
ADHD [63]	1	8	Patients	8–46 years	3 females	0	9 min session	Self-assessments	Positive improvement in symptoms
Anxiety [92]	1	15	Patients	18–64 years old	10 females	0	20 min sessions	Neuropsychological assessment tests—SIGH-A, CGI-S, CGI-I, PSQI	Treatment was effective and well tolerated. Reduction in anxiety symptoms
Bipolar disorder [58]	1	5	Patients	60–85 years	3 females	1	10 min session	Neuropsychological assessment tests—YMRS, TMT-B, PHQ9, DMS	Improvement in cognitive tasks (e.g., cognitive flexibility, impulsivity, and attention), except for verbal fluency
Motor performance [70]	1	56	Healthy volunteers	18–30 years	42 females	1	5 min session	Motor performance—finger tapping test	Transcranial light irradiation may improve the motor performance in healthy subjects. Safe with no physical tissue damage
Schizophrenia [71]	1	32	Patients	49.88 mean age	32 not specified (1 article)	1	15 min sessions	Neuropsychological assessment tests—MMES and PANAS	No significant improvements
Severe disorder of consciousness [62]	1	8	Patients	54.1 mean age	5 females	0	10 min sessions	Neuropsychological assessment tests—r-CRS	Increased alertness and awareness of the chronic DOC patients
Sexual dysfunction [83]	1	20	Patients	18–65 years	11 females	1	30 min	Self-assessments. Neuropsychological assessment tests—SAFTEE-SI	Demonstrated a significant therapeutic effect, with reversal of sexual dysfunction in patients with multifactorial causation
Traumatic brain injury with depression [59]	1	39	Patients	40.5 mean age	20 females	0	30 min sessions	Neurological assessment tests—HDRS, QIDS	Efficacy in depression symptoms. Some patients responded within 4 weeks, more rapid than the response typical of standard oral antidepressants
Blood conditions [32]	1	90	Patients	76.2 mean age	41 females	1	30 min sessions	Blood tests	Improvement in blood lipid and hemorheology behaviour of patients with vascular disease
Vertebrobasilar insufficiency [68]	1	25	Patients	64 mean age	20 females	0	NR	Diagnostic test—De Klyn’s test, and balance test—Berg Balance Scale	Improvement in global stability and balance, along with reduction of VBI symptoms, better blood perfusion and an increased level of oxygen in brain tissue

*BRIEF-A* Behaviour Rating Inventory of executive function-adult, *CFT* category fluency test, *KBIT* Kaufman brief intelligence test, *SDAG* ADHD rating scale for parents from the Italian Scala per i Disturbi di Attenzione/Iperattività per Genitori, *ASRS* Adult Attention-Deficit/Hyperactivity Disorder Self-report Scale, *AST* Alberta Smell Test, *ABM* Attention Bias Modification, *APSI* Autism Parenting Stress Index, *ANAM* Automated Neuropsychological Assessment Metrics, *BDAE* Boston Diagnostic Aphasia Exam, *BNT* Boston Naming Test, *CVLTII* California Verbal Learning Test II, *CARS* Childhood Autism Rating Scale, *CGDS* Chinese Geriatric Depression Scale, *CDR* Clinical Dementia Rating Scale, *CGI-I* Clinical Global Impressions-Improvement, *CGI-S* Clinical Global Impressions-Severity, *CPT* Clock Copying Test, *CDT* Clock Drawing Test, *r-CRS; 0–23* Coma Recovery Scale, *CTHEV* computer test for hemispheric emotional valence, *CES-D* Depression Scale, *DCS* diffuse correlation spectroscopy, *DVT* digit vigilance test, *ADAS-Cog* Disease Assessment Scale-Cognitive, *FAQ* Functional Activities Questionnaire, *GAS-10* Geriatric Anxiety Scale-10 Item Version, *GAF* Global Assessment of Functioning, *HARS* Hamilton Anxiety Rating Scale, *HDRS* Hamilton Depression Rating Scale, *HSQ-ASD* Home Situation Questionnaire-ASD, *HKLLT* Hong Kong List Learning Test, *HADS* hospital anxiety and depression scale, *IADL* Instrumental Activities of Daily Living, *LVFT* lateral visual field test, *LMT-I and II* logical memory test-immediate recall, *DMS* match-to-sample, *MMSE* Mini‐Mental State Exam, *MEFT* Modified Eriksen Flanker Test, *mRS* Modified Rankin Scale, *MERS-R* Montefiore Einstein Rigidity Scale-Revised, *MoCA* Montreal Cognitive Assessment, *NIHSS* National Institutes of Health Stroke Scale, *OCS* opioid craving scale, *PGNG* Parametric Go/No-Go level-1, *PHQ9* Patient Health Questionnaire-9, *PNT* Philadelphia Naming Test, *PSM* Physical Self Maintenance, *PSQI* Pittsburgh Sleep Quality Index, *PSQI* Pittsburgh Sleep Quality Index, *PANAS* Positive and Negative Affect Scale, *PVT* psychomotor vigilance task, *Q-LES-Q* quality of life enjoyment and satisfaction questionnaire, *QIDS-C* Quick Inventory of Depressive Symptomatology-Clinician Rating, *RTT* Reaction time test, *Rey-O* Rey-Osterrieth Complex Figure Test, *SRS-2* Social Responsiveness Scale-2nd edition, *SCID* Standard Clinical Diagnostic Interview, *SIGH-A* Structured Interview Guide for the Hamilton Anxiety Scale, *SAFTEE-SI* Systematic Assessment for Treatment-Emergent Effects-Specific Inquiry, *QIDS* The Quick Inventory of Depression Symptomatology-Self Report, *TUG* timed up-and-go, *TMT-A and B* Trail making test A and B, *T-SRQ* Transcranial LightTherapy Self-Report Questionnaire, *UPDRS* Unified Parkinson Disease Ratile, *WAISIV* Wechsler Adult Intelligence Scale IV, *WCST* Wisconsin Card Sorting Task, *WMQ* Working Memory Questionnaire, *YMRS* Young Mania Rating Scale. Outcomes measured highlighted in bold refer to the most commonly used, if there was one, and the underline refers to the category/type of outcomes

## Discussion

### Device description and analysis

Although it does not seem to exist a clear tendency towards the use of a particular device for a particular application, some advantages and disadvantages from each category can be described.

#### Laser handpiece

Laser handpieces were the most featured devices across all records. These devices are simple and easy to handle by the operator, which can quickly and conveniently place the device in any area of the head. The small actuation area of these devices also allows a more precise treatment in the target location. On the downside, in most situations, the assistance of the operator is expected to correctly identify the desired site of actuation, as well as to position the handpiece in the correct place and to hold it during treatment, changing locations whenever needed. This implies that the assistance of a specialized third party is always required, which may affect the patient’s ability to perform treatments as needed, and the operator will be completely occupied by the task since they have to hold the handpiece.

Overall, the results of the studies using these devices showed an improvement of the symptoms for various diseases and positive findings in physiological characterization and cognitive function studies, except for two studies targeting stroke, that used NeuroThera® Laser System, in which there were no significant improvements in patients’ condition [55, 57]. The most used device was the Model CG-5000 Laser, with 19 reports, which was mostly used in physiological characterization and cognitive function studies. This device works on the highest range of wavelength covered in this review, 1064 nm, and it was used in continuous mode with a power output up to 3.5 W. Studies that use these parameters showed safety, negligible heat, and no physical damage [20, 29]. One study reported that the neuromodulation caused by PBM was not due to the heat generated during treatments [48]. Nonetheless, further studies are warranted to corroborate this finding. The reason why this device was frequently employed could be due to its wavelength, since reduced light scattering in the head tissues was demonstrated with this wavelength [10, 41, 51, 58].

#### LED clusters

LED clusters were the second most recurrent devices in the revised records, relating to six different devices. Generally, the actuation area of these devices is larger, which is useful for stimulating a greater area, being also more forgiving in placement. However, this can also be perceived as an issue when the stimulation of a smaller area is required. Usually, these devices use more than one LED cluster simultaneously, meaning that several regions of the brain can be stimulated at the same time.

Although these devices presuppose the assistance of an operator to hold them in place, several articles report the use of mechanisms, such as fabric covers, to keep the clusters in position during treatment and, thus, freeing the operator during the stimulation session, as opposed to the laser handpieces [18, 19, 30]. These devices were used for different health conditions, such as TBI, stroke, depression, Gulf War illness, and cognitive function deficits, showing positive improvements in cognitive function, as well as antidepressant effects, neuromodulation, and overall improvement of neurological symptoms. The most used LED cluster was the MedX Health Model 1100, relating to 6 reports. The most distinctive feature of this device is its versatility since it encompasses two or three LED clusters that can be applied simultaneously in different locations (e.g., its use for ear stimulation). It uses red (633 nm) and NIR (870 nm) light, broadening the possible stimulatory effects since articles report the efficiency of both wavelengths [3, 33].

#### Helmets

The helmet configuration presents another advantage that is lacking in the previous categories of devices; since the placement of the helmet is straightforward, it enables home use and autonomy of the patient. However, the device may fit differently in different patients, missing the intended areas for stimulation. One other advantage that may arise from this type of device is that distinct areas of the helmet can be turned on, at different times, to enable customized treatments. Nevertheless, only one article referenced the use of this feature, meaning that, although a possibility, it is not considered of significance in the use of such devices [14].

The application of helmet devices is associated with great power output values, which may cause overheating of the head tissues. Most articles report the use of a cooling system, usually with fans, to prevent this situation [39, 73, 80, 96]. For LED helmets, all articles showed improvements in many health conditions. Regarding the laser helmet, its use was not conveniently justified, and it does not appear to provide any advantage compared to LED helmets. Furthermore, the article in which this device was used investigated schizophrenia and showed no significant improvements in this condition [71]. The Cognitolite Transcranial Photomodulation System—a modular helmet comprised of several LED clusters, and the Thor photomedicine LED lined helmet, which is a metal structure encrusted with LEDs, were the most commonly reported LED helmets.

#### Localized helmets

Localized LED helmets relate to a specific type of device by the company Vielight®. The advantages of using these devices are their home use and simple positioning, similar to other helmets, but with the addition of a simple and lightweight design, and an intranasal LED. Even though this device was designed and is commercially available to enhance mental performance, it was used in 12 reports for eight different purposes, namely AD [105, 106], autism [99], cognitive function improvements [100, 104], dementia [26], Gulf War illness [101], PD [23], physiological characterisation [102], and TBI [103]. Overall, all articles showed clinical improvements, except one for cortical excitability, where the lack of success was attributed to the use of healthy and young subjects, as opposed to other similar studies which showed positive findings in older or neurological diseased subjects [102]. Also, another study focused on the improvement of TBI symptoms showed positive outcomes in only one subject, however, the authors state that it may be due to a placebo effect [103].

A downside of using these devices may be the specific locations of the LEDs and the inability to move the actuators as desired, which can limit their use for certain health conditions if the affected area of the brain to be stimulated does not coincide with the location of the LEDs.

#### LED headband

The LED headbands, which were used in a study dedicated to the improvement of cognitive function [93] and another for the improvement of anxiety symptoms [92], may be useful to stimulate wider areas. However, their design appears to be more focused on the forehead. Furthermore, one of the devices, the OEG-SpO2, was designed to be portable and can be placed by the user, which allows home use. Both reports showed positive findings in the scope of their studies [93].

#### Laser needles

The laser needles device by Weber Medical comprises four laser needles, each with a very small actuation area. In theory, this system allows the simultaneous stimulation of different locations, according to the placement of the needles. Nevertheless, the study that reported using this device placed the needles close together. This device encompasses a fixation system that frees the operator during the stimulation session, but it is a complex system to be applied at home by the patient alone. Also, the setup of this device is one of the most complex, as it requires the placement and fixation of each needle individually. Changing the stimulation site, for instance, will require more time compared to the other devices, which are simply placed in the actuation site. The study that used this device found neuroplastic changes after its use [72].

#### Intranasal

There were seven reports of intranasal devices used for brain PBM. One report relates to a light penetration study that used a laser intranasal device [37]. A study with demented patients used a Vielight® intranasal device as a complementary home treatment after sessions of PBM therapy in the clinic [26]. Regarding intranasal devices used individually, a study of blood conditions [32] used the BLOODCARE Medical Laser Device, and a PBM study with PD patients [33] used an intranasal LED. The remaining three reports concern simultaneous use of intranasal and transcranial devices for the treatment of Gulf War illness [14] and AD [25].

The main advantage of this type of device is the stimulation of the inner parts of the brain. These devices are portable and easy to use, however, they can be considered invasive. The Vielight® devices Neuro Alpha and Neuro Gamma include an intranasal LED, which was used in all 12 reports of these devices. The results of the studies which combined intranasal devices with transcranial devices cannot clearly be related to the use of intranasal devices solely, since studies for the same pathologies with only transcranial stimulation showed similar results, namely for the treatment of dementia [94], AD [24], and Gulf War illness [101]. However, solo intranasal devices led to improvements in signs of PD [33] and blood hemorheology behaviour [32].

From the 15 articles that described the simultaneous stimulation of transcranial and intranasal, eye, or ear stimulation, there is no clear difference arising from their combination, since similar studies also showed positive results when stimuli were applied individually. Thus, further studies which directly compare the sole use of intranasal, ear, and eye stimulation with their concomitant utilization with transcranial devices are warranted to determine its efficiency and necessity.

Moreover, there was no significant trend toward the use of LEDs or lasers since they were used in similar numbers, and there is no consensus if either one is preferable to the other. Cost may be a decisive factor as lasers are generally more expensive [107].

Table 3 presents an overview of the design, advantages, and disadvantages of each category of device, along with the most used device in each category.

**Table 3:** Comparison of device categories

Device category	Design	Advantages	Disadvantages	Most commonly used
Laser handpiece	Control unit and a fibre optic cable that connects to the handpiece	Versatile; easy to handle; quickly placed; simple construction; precise treatment	Operator dependent	Model CG-5000 laser
LED cluster	Control unit and LED cluster (usually flat) with higher number of LED diodes, or a single component containing the LEDs and control unit	Wide actuation area; solutions to fix the device; multiple simultaneous placements	No precise treatment	MedX health model 1100
LED helmet	LED clusters arranged in a helmet shape; LEDs incrusted in a metal or plastic structure; or neoprene pads filled with LED rows	Straightforward placement; home use; autonomy; simultaneous stimulation of different areas	May not fit different head sizes; higher power—requires cooling systems	Cognitolite transcranial photomodulation system and the thor photomedicine LED lined helmet
Laser helmet	LEDs incrusted in plastic structure, shaped like a helmet	Straightforward placement; home use; autonomy; simultaneous stimulation of different areas	May not fit different head sizes; higher power—requires cooling systems	–
Localized LED helmet	Two metallic straps placed on the top and crown of the head, with three posterior transcranial LEDS, one anterior transcranial LED, and one intranasal LED	Home use; simple positioning; light weight; with intranasal	Specific locations of the LEDs—can limit use for certain health conditions	Vielight® Neuro Gamma and Vielight Neuro Alfa
LED headband	Headband with sensors and LEDs and a plastic cover to disguise the mechanical components. Connected to a portable control unit	Wide actuation area; home use; portable	More suited for forehead; NIRS device	OEG-SpO2 and Cerebral Science
Laser needles	Four stainless steel laser needles connected to a control unit through fibre optics. Held in place with wire holders attached to a crown	Fixation system; may allow simultaneous stimulation of different locations	Time consuming; complex fixation system; small actuation area	Laser WeberMedical Klasse 1, type BF, laser class 3B
Intranasal LED	Single LED with control unit	Simple; light weight; portable; stimulation of interior brain	More invasive, more structures to penetrate	Vielight® intranasal 810
Intranasal laser	Single laser with control unit	Simple; light weight; portable; stimulation of interior brain	More invasive, more structures to pass through	BLOODCARE medical laser device

### Parameter analysis and condition relation

One of the purposes of this review was to understand if there were trends for the treatment of certain health conditions with brain PBM. It should be noted that the eight parameters initially established for the review were selected because they relate to a full characterisation of the stimulation, which is provided in 37 of the reviewed articles. However, it is possible to fully define the stimulation parameters with only the actuation site, area of actuation, power output, session time, mode of operation and wavelength. The purpose of comparing the reports for each of the eight parameters was to demonstrate the inconsistency of information, which often leaves the stimulation underdefined, and the need for standard methods of reporting PBM studies. Even though there are no clear indications for a standard treatment procedure for each condition, some trends can be pointed out.

Overall, the results of studies showed positive findings following the use of PBM, with no relevant difference due to the subjects’ sex, but a possible difference according to age and condition, a hypothesis raised from a study of cortical excitability in healthy volunteers [102].

#### Location of stimulation

Regarding stimulus location, the PFC is the most commonly stimulated region of the brain, with some articles mentioning the lack of hair in the forehead, and therefore, higher light penetration [39, 66, 76]. However, a substantial number of studies (e.g., those which used helmet devices) stimulated the scalp with hair and also showed beneficial outcomes, which means that the effect of hair in light penetration in head structures should be further investigated to understand if it has a significant effect. Furthermore, another advantage of the PFC as the PBM target location is that this region has a relevant role in the processing of simultaneous stimuli and thought, and in cognitive control, which explains its prevalent use in mental and physiological conditions [85, 108].

As previously mentioned, it was common for several regions of the brain to be stimulated simultaneously, especially in degenerative diseases and/or conditions, such as AD, PD, and dementia. For dementia and AD, which are associated with overall damage in cerebral structures and loss of neuronal communication, it appears reasonable that the whole head should be stimulated, since the damage is spread through the whole brain [109]. Regarding PD, deeper brain structures, such as the basal ganglia and hypothalamus, and back structures, such as the cerebellum and the brainstem, are affected, which justifies the broad placement of the stimulation since the aim is to reach deeper areas of the brain [110]. It is relevant to understand if the transcranial light can reach these depths, since a cadaver study demonstrated that light only penetrates up to 40 to 50 mm of the brain [38]. One study mentions the use of intraoral stimulation to better reach these internal structures, which may be a solution for this issue [28]. Additionally, Gulf War illness and schizophrenia also showed a trend in the stimulation of several regions, but the number of articles is reduced and, thus, this trend may be biased.

#### Wavelength

Concerning wavelength, 810 nm is the most widely used across all health conditions, with fluctuations from 808 to 850 nm. When red light is used, it is usually combined with NIR light, except in a LED helmet applied in PBM for PD patients [16], a LED device lacking a detailed description which was used to assess the effect of PBM in non-demented elderly women [79], and an intranasal LED device employed to treat generalized anxiety disorder [33], in which only red light was used for the stimulation.

The rationale behind the use of this wavelength is justified by its reduced absorption by the three major tissue chromophores (i.e., haemoglobin, melanin, and water), showing also effective absorption by the mitochondria, which is currently believed to be one of the mechanisms responsible for the effects of PBM on the brain [1–3, 77].

Nonetheless, a higher wavelength, namely 1064 nm, is more commonly used in physiological studies, specifically for cognitive function improvement and physiological characterisation, and in studies of fear and bipolar disorder. The use of this longer wavelength is associated to a deeper a penetration through the various head tissues. This characteristic is attributed to reduced scattering of photons in this wavelength. Studies show that even though this wavelength is not optimal for the known mitochondrial process behind the positive results of PBM, it still is produces this biological response [11, 111–113]. Additionally, there are some indications in literature that there is another mechanism activated by higher wavelengths, at light sensitive ion channels, but further research is required to establish the relevance and effectiveness of this mechanism [2]. Even so, the fact that this wavelength can reach deeper into the brain structures compensates for its lack of effect at the mitochondria, and some studies believe that this trade-off is actually beneficial and increases the positive results [10, 41, 51, 58].

#### Mode of operation

In the reported PBM studies for physiological characterization, light in continuous mode was the most used, whereas in studies of PBM in degenerative brain diseases, light in pulsed mode was more common. Among the included articles (n = 97), one study in AD patients compared both continuous and pulsed stimulation, observing that continuous stimulation caused a significant and large enhancement of neural activity in the gamma band [89]. One article assessing the effect of PBM in cortical excitability studied the use of pulsed NIR light in the 5 Hz, 10 Hz and 20 Hz frequencies, with a 50% duty cycle. This study noticed that not only low energy NIR stimulation changed the EEG signal when compared to the control group, but also that increasing frequency had a greater impact on brain activity, which may have a role in the improvement of memory [76].

Another article, which did not fit the initial inclusion criteria, compared the use of continuous or pulsed light in animal and human studies and concluded that pulsed light is more beneficial, especially for wound healing and post-stroke management [114]. Considering that there is no consensus on the mode of operation for the remaining health conditions, further studies are necessary to determine which mode of operation is more adequate for different circumstances.

#### Power density and power output

Power density was usually used at around 250 mW/cm^2^, especially in physiological conditions. Lower values were also applied, the lowest being 4 mW/cm^2^ for ear stimulation in a PBM study with Gulf War illness patients [14]. Studies investigating PBM in TBI, dementia, anxiety, severe disorder of consciousness and blood conditions explored power density values below 50 mW/cm^2^.

The highest recorded power density value was 800 mW/cm^2^, which was applied with a laser helmet, and a frequency of 75 Hz (20% duty cycle), in a study of PBM in schizophrenia patients [71]. Despite the high-power density values, no study reported negative side effects because of heat generated by light stimulation. As previously stated, helmet devices, which usually presented higher power values, were often combined with a cooling system to prevent this issue. High power values (10 to 13 W) were used in a PBM study with patients with TBI and related depression. This study utilized the laser handpieces LiteCure® LT1000 and Diowave 810 with a sweeping motion to avoid overheating, and patients reported a comfortable warm feeling during the sessions [59]. A study of the effects of PBM in AD patients used a laser handpiece Aspen diode laser, with power values between 5 and 25 W, oscillatory motion across the treatment site, fans, and pauses in treatment to maintain normal skin temperature [67]. These instances bring into discussion whether PBM treatments with higher power outputs can overheat the head tissues and cause damage. Even though these devices used cooling systems, it would be crucial to determine a safe threshold for power output per area (i.e., power density) to define a maximum power output value for future studies.

Table 4 presents a more detailed overview of the trends previously mentioned. The health conditions were subgrouped by category, namely degenerative, mental, physiological conditions, brain injury, or others, and the parameters provided are location, wavelength, mode of operation and power density, which are the most commonly reported, along with treatment time, which was already reported in Table 2. For each parameter and condition, the mode value is indicated. In cases where there were two modes or two values had close frequencies, both values are presented. NR refers to unreported values, which were considered in this analysis since, in several parameters, this was a significant number. When NR was the mode, the next value with greater frequency is also presented, when possible. When there was not a mode value, the whole range of values was presented. Energy density, energy per session and power output values were often not reported by the studies, and thus not considered.

**Table 4:** Target condition and parameter mode

	Target condition	No. of reports	Location	Wavelength (nm)	Mode of operation	Power density (mW/cm^2^)	Device
Brain injury	Traumatic brain injury	12	Several regions* (75%)	629–633 (42%);	Continuous (42%)	6.4–100 (75%)	Helmet/MedX Health Model 1100
	Traumatic brain injury with depression	2	PFC and temporal region (100%)	810 (100%)	Continuous and pulsed 10 Hz (100%)	NR (100%)	LiteCure® LT1000
	Stroke	4	Several regions* (100%)	808 (75%)	NR (75%); continuous and pulsed 146 Hz (25%)	NR (75%); 22.2 (25%)	NeuroThera® Laser System
Degenerative	Alzheimer’s disease	8	Several regions* (100%)	810 (63%)	Pulsed 10 Hz (50%)	23.1–354 (100%)	Helmet/localized helmet
	Dementia	4	Several regions (100%)	810 (75%)	Pulsed 10 Hz (50%); NR (50%)	14.2–41 (100%)	–
	Parkinson’s disease	6	Several regions* (67%)	810 (33%); 904 (33%)	Pulsed 50 Hz (33%)	6–100 (50%); NR (50%)	–
Mental	Anxiety	1	PFC and ventrolateral PFC (100%)	830 (100%)	Continuous (100%)	30 (100%)	LED not described
	Bipolar disorder	1	PFC (100%)	1064 (100%)	Continuous (100%)	250 (100%)	CytonPro
	Depression	4	PFC (100%)	808–830 (75%)	Continuous (100%)	33.2–700 (100%)	–
	Fear	2	PFC (100%)	1064 (100%)	Continuous (100%)	250 (100%)	Model CG-5000 laser
	Anxiety and depression	2	PFC (100%)	810 (50%); 945 (50%)	Continuous (50%); NR (50%)	250 (50%); NR (50%)	–
	Autism	3	Several regions* (67%)	810 (67%)	Pulsed 10 Hz (33%); pulsed 40 Hz (33%)	100 (transcranial); 75 (anterior transcranial); 25 (intranasal) (67%)	Vielight® neuro alpha/gamma
	Schizophrenia	1	Several points (100%)	630 and 810 (100%)	Pulsed 75 Hz 20%DC (100%)	800 (100%)	Laser helmet
	ADHD	1	PFC (100%)	660 and 905 (100%)	Superpulsed (100%)	NR (100%)	Theralase® TLC-2000 CLT
Physiological	Motor performance	1	Motor cortex (100%)	808 (100%)	Continuous (100%)	200 (100%)	Laser not described
	Physiological characterisation	21	PFC (mostly right side) (71%)	808–850 (48%); 1064 (43%)	Continuous (71%)	250–285 (43%)	Model CG-5000 laser
	Cognitive function	18	PFC (mostly right side) (83%)	1064 (50%); 800–850 (44%)	Continuous (72%)	250 (44%)	Model CG-5000 laser
	Blood conditions	1	Intranasal (100%)	650 (100%)	NR (100%)	8.38 (100%)	BLOODCARE medical laser device
Other	PTSD (Gulf War illness)	6	Several points* (100%)	810–850 (67%)	continuous (67%)	4–100 (100%)	–
	Severe disorder of consciousness	1	Upper edge of two fossa sphenoidalis (100%)	785 (100%)	NR (100%)	10 (100%)	Power twin 21
	Sexual dysfunction	1	Dorsolateral PFC (100%)	823 (100%)	NR (100%)	36.2 (100%)	Omnilux new U device
	Drug abuse	3	Dorsolateral PFC (100%)	810 (100%)	NR (67%); pulsed 10 Hz (33%)	250 (100%)	LED not described
	Vertebrobasilar insufficiency	1	Back of neck (100%)	810–830 (100%)	Pulsed 6 kHz (100%)	NR (100%)	CTL-1100 low power laser

*Several regions detailed: TBI—Circle of Willis, over the mastoid processes, PFC, entorhinal cortex, hippocampus, frontal, parietal, temporal, occipital, and temporoparietal regions, default mode network (DMN), salience network (SN), other not specified; stroke—PFC, frontal, temporal and occipital regions, vertex of the head, Wernicke’s area, around ears, other not specified; AD—PFC, frontal and temporal regions, enthornial cortex, hippocampus, and intraocular; dementia—PFC, frontal and temporal regions, entorhinal cortex, hippocampus, and other not specified; PD—PFC, frontal and temporal regions, entorhinal cortex, hippocampus, basal ganglia and substantia nigra (intraoral), back of neck, other not specified; autism—PFC, frontal and temporal regions, entorhinal cortex, hippocampus; Gulf War illness—PFC, frontal and temporal regions, entorhinal cortex, hippocampus, ears

**Fig. 4:**
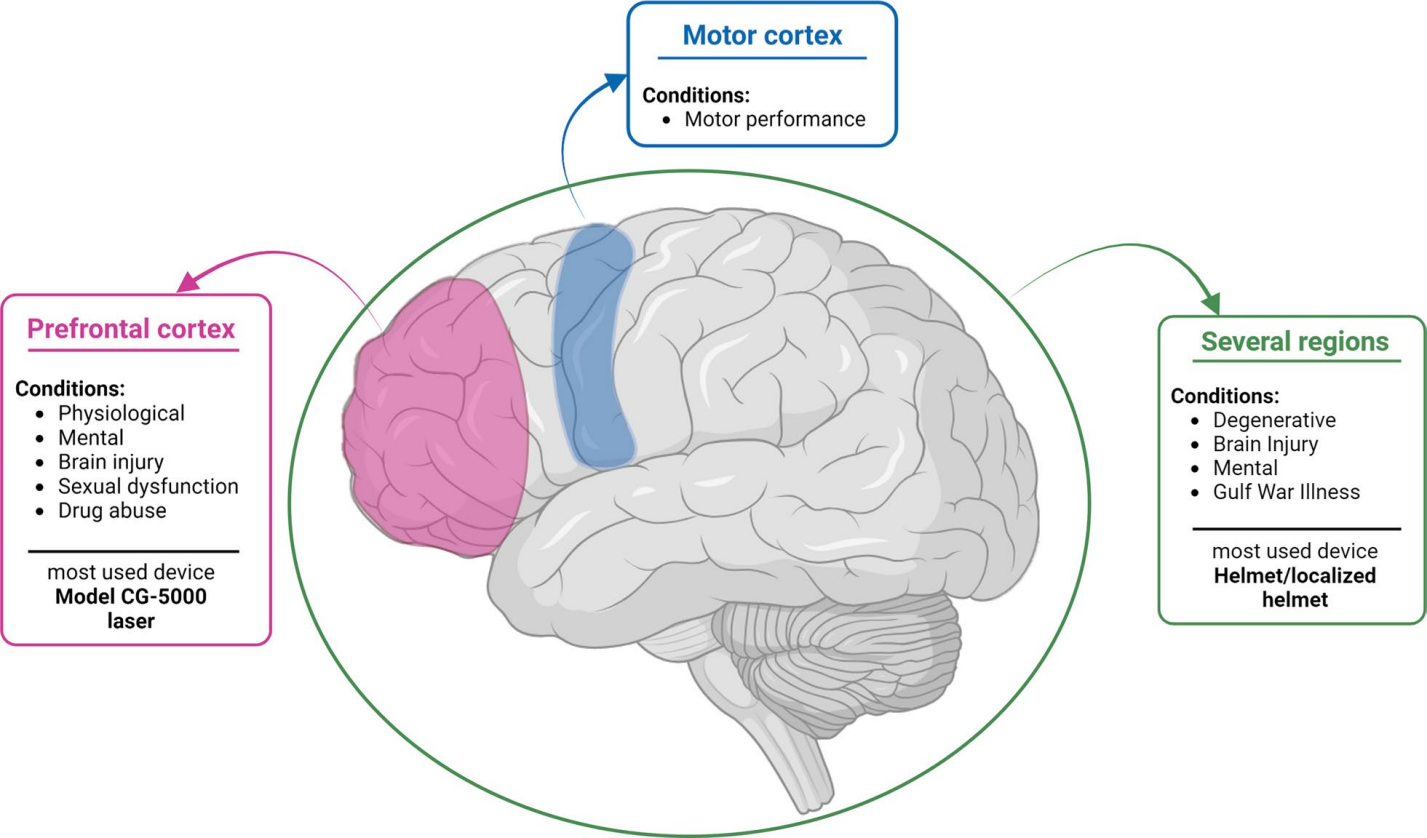
Location of stimulation, condition, and device overview. Created with BioRender.com

Figure 4 depicts the brain regions stimulated, and thereby affected, by each type of condition. In some studies, the brain region being stimulated was not clear, and thus these cases were not included.

### Light penetration

Light penetration studies were focused on the wavelength and power values that enable more efficient light delivery to the targeted brain areas. One study detailed the use of continuous and pulsed light but did not show different results based on different modes of operation [38].

The wavelength used in studies assessing light penetration ranged between 633 and 970 nm, and power density between 35 and 700 mW/cm^2^. Light at 700 mW/cm^2^ and with 800 nm wavelength, can penetrate the skull, but with an attenuation greater than 95% [36]. Despite this power density value being higher than the values commonly reported in the articles under review, it has been proved to be safe for clinical use in major depressive disorder [22]. Furthermore, one study of PBM in schizophrenia patients used a higher power density value, namely 800 mW/cm^2^, and did not show significant improvement in cognitive impairment. However, the authors noted a decline in depression and anxiety factors in neuropsychological tests, with no apparent negative side effects, namely overheating and tissue damage, from the use of a high power density value [71]. Studies in cadaveric heads show penetration of 808 nm light through the scalp, skull, and meninges to a brain depth of approximately 40 mm [38].

Moreover, a study delivering intranasal light stimulation to a cadaveric head demonstrated that transsphenoidal delivery of light to brain structures is possible [37].

An additional study that did not fit the inclusion criteria (it used lamb skulls) showed that approximately 2.9% of the 810 nm continuous light delivered at a power of 10 to 15 W penetrated 3 cm of tissue (skin, skull, and brain), as opposed to 980 nm light in the same circumstances, of which only 1.2% penetrated the same distance in the lamb brain, remarking the impact of wavelength in this phenomenon [115].

Considering wavelength, as stated in Sect. 4.2, some studies justify the use of infrared light of 1064 nm because it is less scattered in the head tissues when compared to lower wavelengths, allowing deeper tissue penetration (i.e., around 30 to 40 mm), of 1–2% of the light treatment [10, 41, 51, 58]. However, some simulation studies, which do not fit the inclusion criteria, but contain useful information in the discussion of the penetration of light through head the tissue, have disagreeing information on this topic. One Monte Carlo optical simulation study demonstrated that photons of 810 nm and 660 nm wavelengths reached wider and deeper into the brain structures, when compared to 1064 nm [116], while another Monte Carlo study concluded that 1064 nm photons penetrate deeper than 810 nm photons, due to less scattering by the tissues. However, it also pointed out that higher melanin contents in the scalp may affect the 1064 nm photons penetration, favouring the 810 nm photons [117]. To address the scattering of photons through the head tissues, an additional Monte Carlo study conducted a simulation with multiple, evenly distributed emitters across the scalp (working under the safe thermal limit), to understand if it could be possible to increase photon density in the brain. The study revealed that this configuration significantly increased photon flux and distribution in the brain. This study used 850 nm NIR, which further implies that the scattering issue of wavelengths lower than 1064 nm could be resolved [118].

**Fig. 5:**
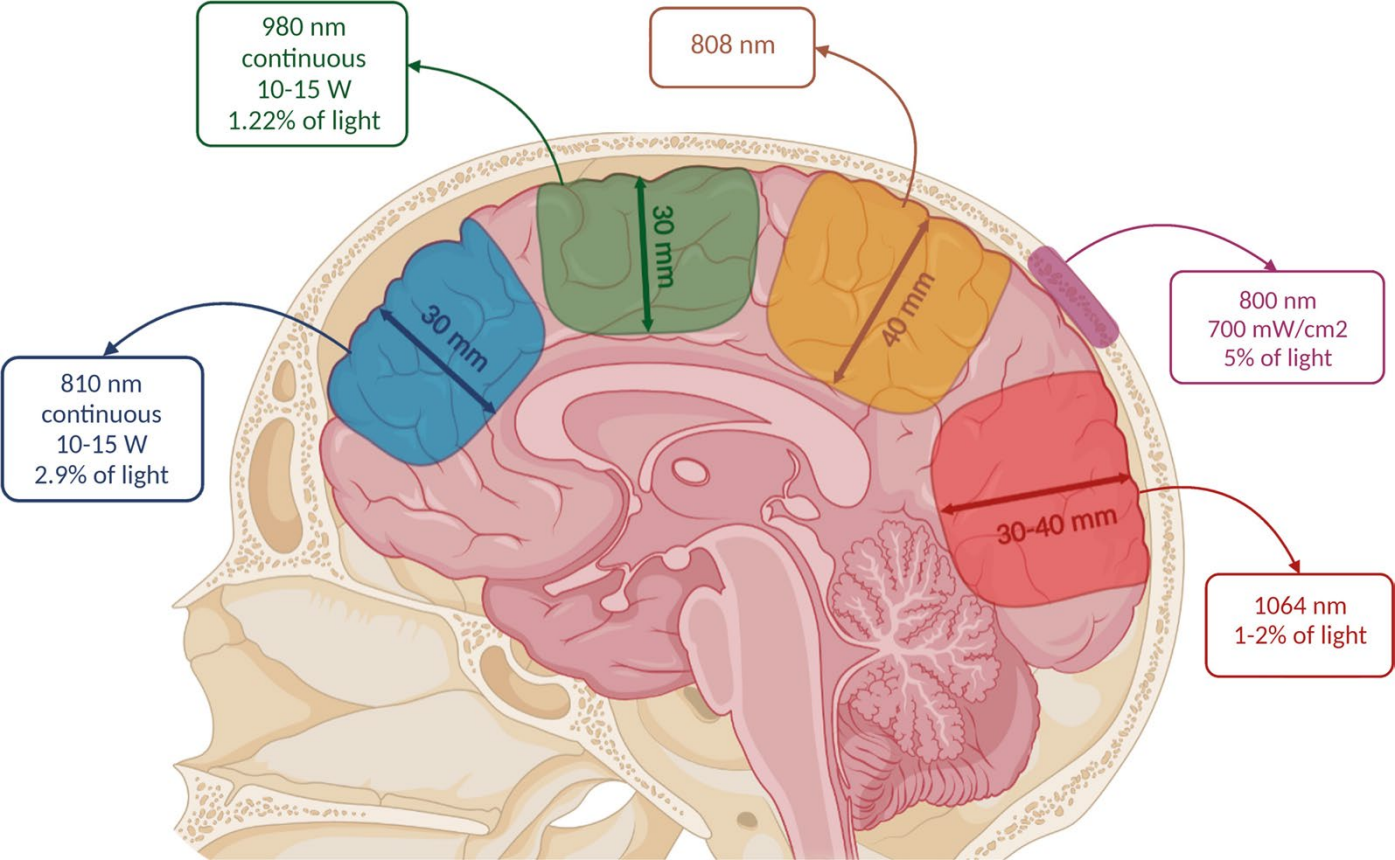
Illustration of light parameters and respective penetration in head tissues. The depicted locations are not related to the parameters but are approximations for better visualization. Created with BioRender.com

Figure 5 is the summary of the information on light penetration through the brain collected from the abovementioned studies, not including simulation results. The depth of light propagation is represented as a function of the light parameters used in the respective experiments.

Although light penetration through the head structures plays a dominant role in current studies of PBM for neurological conditions, there are indications in literature that there may be indirect or abscopal effects associated with stimulation with red and NIR light [1, 3]. A 2018 study could even find a relation between PBM applied to the back and thighs, and the improvement of depression symptoms [119]. Studies with animals further emphasise this effect, specifically one example which studied PD with mice models, applying NIR radiation only to the body and protecting the head with aluminium foil, and still seeing protective effects in brain cells [120].

These studies suggest that the circulatory system might play a role in the transmission of the mechanisms activated in a location away from the target tissue, by the NIR stimulation, allowing these mechanism to be reflected in the tissues of interests [3, 120, 121]. Nevertheless, more research is warranted to understand this effect and what purpose it serves in PBM of the brain.

### Limitations

The lack of clear reporting of PBM parameters is one of the limitations of the present review. Wavelength was one of the inclusion criteria, and the location of the stimulation was mentioned in all articles, except for one [60]. Besides these two parameters, much of the PBM information was left out in several studies. For instance, the mode of operation is a fundamental aspect of the stimulation since several studies showed that can impact the effects of the treatment, but it was only mentioned in 25% of reports. Also, the lack of information on the area of actuation, power/energy density or power output impairs the fully characterization of the amount of light that is applied to the head. Additionally, from the 57 devices found in this research, 28% were not described in terms of design, besides identifying the use of LEDs or lasers. Even for those devices that were described, the lack of visual support occasionally hindered the understanding of the device design and functioning. The acquisition and categorisation of this information was difficult in these circumstances, which compromised the comparison of devices, parameters and the effectiveness of the stimulation protocols.

Regarding the number of subjects, 25% (n = 97) of studies have 10 or less participants. Along with this, the methods to determine the efficiency of treatments rely mainly on neurophysiological tests, which are often not sham-controlled, raising questions about placebo effect in such studies [17, 25, 26, 33, 61–64, 82, 87, 101]. On the other hand, 77% of the studies addressing physiological characterisation and cognitive function improvement were sham-controlled. More specifically, physiological characterisation studies often used imaging techniques to accurately determine differences in oxygenation, implying that the results of studies in these areas are more reliable [11, 47, 72, 76, 102].

Comparative studies for different parameters are required to establish the most adequate regimen for different circumstances (e.g., patients, diseases). Different articles assessing the effectiveness of PBM for treating the same pathology/medical condition used distinct combinations of parameters and showed positive outcomes. This hinders the conclusion of which stimulation protocols are the most adequate to achieve the greatest benefits.

## Conclusions

The aim of this review was to analyse and understand the devices that are being used for brain PBM, as well as their operating parameters, in order to determine the most appropriate characteristics and parameters for certain neurological conditions. Similarly, this review envisioned the evaluation on which device configuration presents more promising results. The inclusion criteria were broad to include as many records of devices employed in brain PBM as possible, rather than limiting to a specific group of devices to obtain a more comprehensive perspective.

Although brain PBM has been studied for over two decades and has been proven to induce beneficial effects on various health conditions, it is uncertain which parameters are more suitable and cause more beneficial effect for different pathologies and patients. This fact is further intensified by the lack of consistency in the reporting of these studies, both in terms of parameters and device description, which is detrimental for research of this field since a significant part of the studies and results cannot be reproduced neither compared. Thus, the actuation site, area of actuation, power output, session time, mode of operation and wavelength should be always provided, so that the stimulation can be well-defined and replicated by others. Moreover, further comparative and sham-controlled studies are demanded to compare parameters and the respective results, establishing more reliable conclusions.

The correlation between devices/parameters and health conditions has been discussed with the purpose of finding some trend. PBM studies addressing physiological characterization and cognitive function improvements are those who display more defined trends, namely the use of higher wavelength, PFC as the stimulation site, power density values around 250 mW/cm^2^, and the use of continuous mode of operation. For neurodegenerative diseases, the tendencies noted were the use of 810 nm light, multi-spot stimulation, and the use of pulsed light. The PFC was often stimulated in mental conditions. Besides these trends, there are still no parameters and methods of stimulation that seem more suitable for other health conditions. Regarding the reported devices, it was noticed a preference for laser handpieces. This may be due to their versatility and ease of use. LED clusters appear as an alternative for larger stimulation areas and have fixation options to the head. The use of helmets may be a more suitable alternative for home treatments since its placement is straightforward. Additionally, the customization of treatments with distinct areas of the helmets being turned on should also be studied further.

This review provided insight on the clinical studies of PBM of the brain for neurological health conditions and can possibly serve as reference for future studies in this field. The collection and organisation of information from these studies has resulted in an understanding of which light parameters and types of devices are most commonly used for certain health conditions, however it demonstrates that more research is warranted to establish adequate parameters for future treatments, and that the methods of reporting these studies are lacking coherence.

### Supplementary Information

**Additional file 1: Table S1.** Data base search strategy. **Table S2.** Wavelength (nm) and respective number of occurrences (Fig. 3a data). **Table S3.** Power output (W) and respective number of occurrences (Fig. 3b data). **Table S4.** Power density (mW/cm^2^) and respective number of occurrences (Fig. 3c data). **Table S5.** Energy per session (J) and respective number of occurrences (Fig. 3d data). **Table S6.** Energy density (J/cm^2^) and respective number of occurrences (Fig. 3e data). **Table S7.** Operating mode and respective number of occurrences (Fig. 3f data). **Table S8.** Pulse frequency and duty cycle and respective number of occurrences (Fig. 3g data).

## Data Availability

The datasets used and/or analysed during the current study are available from the corresponding author on reasonable request.

## Abbreviations

AD: Alzheimer’s disease
ADHD: Attention deficit hyperactivity disorder
CVLTII: California Verbal Learning Test II
EEG: Electroencephalogram
fMRI: Functional magnetic resonance imaging
HDRS: Hamilton depression rating scale
IR: Infrared
LED: Light-emitting diode
MRI: Magnetic resonance imaging
MEPS: Motor evoked potentials
NIHSS: National Institutes of Health Stroke Scale
NIRS: Functional near-infrared spectroscopy
NIR: Near-infrared
NR: Non reported
PBM: Photobiomodulation
PRISMA: Preferred Reporting Items for Systematic Reviews and Meta-Analyses
PD: Parkinson’s disease
PFC: Prefrontal cortex
TMS: Transcranial magnetic stimulation
TBI: Traumatic brain injury
VBI: Vertebrobasilar insufficiency
