# Supplementary material for: Devices used for photobiomodulation of the brain—a comprehensive and systematic review
Source: J Neuroeng Rehabil. 2024 Apr 10;21:53. doi: 10.1186/s12984-024-01351-8 (PMC11007916; doi:10.1186/s12984-024-01351-8)
Supplement: Supplementary file 1 — Additional file 1: Table S1. Data base search strategy. Table S2. Wavelength (nm) and respective number of occurrences (Fig. 3a data). Table S3. Power output (W) and respective number of occurrences (Fig. 3b data). Table S4. Power density (mW/cm2) and respective number of occurrences (Fig. 3c data). Table S5. Energy per session (J) and respective number of occurrences (Fig. 3d data). Table S6. Energy density (J/cm2) and respective number of occurrences (Fig. 3e data). Table S7. Operating mode and respective number of occurrences (Fig. 3f data). Table S8. Pulse frequency and duty cycle and respective number of occurrences (Fig. 3g data). [file 12984_2024_1351_MOESM1_ESM.docx]

**Additional Tables**

**Table S1** - Data base search strategy

| Data base | Search Strategy |
| --- | --- |
| PubMed | (((brain[Title/Abstract] OR cortex[Title/Abstract] OR skull[Title/Abstract] OR neural[Title/Abstract] OR head[Title/Abstract]) AND (photobiomodulation[Title/Abstract] OR pbm[Title/Abstract] OR "low level laser therapy"[Title/Abstract] OR lllt[Title/Abstract] OR phototherapy[Title/Abstract] OR "low level light"[Title/Abstract] OR near-infrared[Title/Abstract] OR nir[Title/Abstract])) AND (neuromodulation[Title/Abstract] OR neurotherapy[Title/Abstract] OR stimulation[Title/Abstract] OR transcranial[Title/Abstract] OR intranasal[Title/Abstract] OR intracranial[Title/Abstract] OR oral[Title/Abstract])) AND (device[Title/Abstract] OR actuator[Title/Abstract] OR sensor[Title/Abstract] OR "light delivery"[Title/Abstract] OR laser[Title/Abstract] OR "light emitting diode"[Title/Abstract] OR equipment[Title/Abstract] OR optode[Title/Abstract]) |
| Scopus | ( TITLE-ABS-KEY ( brain OR cortex OR skull OR neural OR head ) AND TITLE-ABS-KEY ( photobiomodulation OR pbm OR "low level laser therapy" OR lllt OR phototherapy OR "low level light" OR near-infrared OR nir ) AND TITLE-ABS-KEY ( neuromodulation OR neurotherapy OR stimulation OR transcranial OR intranasal OR intracranial OR oral ) AND TITLE-ABS-KEY ( device OR actuator OR sensor OR "light delivery" OR laser OR "light emitting diode" OR equipment OR optode ) ) |
| Web of science | brain OR cortex OR skull OR neural OR head (Topic) and photobiomodulation OR pbm OR "low level laser therapy" OR lllt OR phototherapy OR "low level light" OR near-infrared OR nir (Topic) and neuromodulation OR neurotherapy OR stimulation OR transcranial OR intranasal OR intracranial OR oral (Topic) and device OR actuator OR sensor OR "light delivery" OR laser OR "light emitting diode" OR equipment OR optode (Topic) |

**Table S2** – Wavelength (nm) and respective number of occurrences (Figure 3a data).

| **Wavelength (nm)** | **Number of occurrences (counts)** |
| --- | --- |
| [620-660[ | 13 |
| [660-700[ | 5 |
| [700-740[ | 0 |
| [740-780[ | 1 |
| [780-820[ | 47 |
| [820-860[ | 20 |
| [860-900[ | 6 |
| [900-940[ | 6 |
| [940-980[ | 3 |
| [980-1020[ | 1 |
| [1020-1060[ | 0 |
| [1060-1100] | 29 |

**Table S3** – Power output (W) and respective number of occurrences (Figure 3b data).

| **Power output (W)** | **Number of occurrences (counts)** |
| --- | --- |
| [0-4[ | 69 |
| [4-8[ | 4 |
| [8-12[ | 6 |
| [12-16[ | 9 |
| [16-20[ | 0 |
| [20-24[ | 1 |
| [24-28[ | 9 |
| [28-36[ | 0 |
| [36-40] | 1 |
| NR | 33 |

**Table S4** – Power density (mW/cm^2^) and respective number of occurrences (Figure 3c data).

| **Power density (mW/cm^2^)** | **Number of occurrences (counts)** |
| --- | --- |
| [0-30[ | 35 |
| [30-60[ | 17 |
| [60-90[ | 12 |
| [90-120[ | 11 |
| [120-150[ | 0 |
| [150-180[ | 3 |
| [180-210[ | 3 |
| [210-240[ | 0 |
| [240-270[ | 24 |
| [270-300[ | 3 |
| [300-330[ | 3 |
| [330-360[ | 1 |
| [360-480[ | 0 |
| [480-510[ | 1 |
| [510-690[ | 0 |
| [690-720[ | 3 |
| [720-780[ | 0 |
| [780-810] | 1 |
| NR | 17 |

**Table S5** – Energy per session (J) and respective number of occurrences (Figure 3d data).

| **Energy per session (J)** | **Number of occurrences (counts)** |
| --- | --- |
| [0-400[ | 21 |
| [400-800[ | 3 |
| [800-1200[ | 3 |
| [1200-1600[ | 6 |
| [1600-2000[ | 13 |
| [2000-2400[ | 3 |
| [2400-2800[ | 1 |
| [2800-3200[ | 1 |
| [3200-3600[ | 1 |
| [3600-4000[ | 1 |
| [4000-4400[ | 1 |
| [4400-8000[ | 0 |
| [8000-8400[ | 1 |
| [8400-16000[ | 0 |
| [16000-16400[ | 1 |
| [16400-19043[ | 0 |
| [19043-19400] | 1 |
| NR | 57 |

**Table S6** - Energy density (J/cm^2^) and respective number of occurrences (Figure 3e data).

| **Energy density (J/cm^2^)** | **Number of occurrences (counts)** |
| --- | --- |
| [0-20[ | 27 |
| [20-40[ | 12 |
| [40-60[ | 14 |
| [60-80[ | 26 |
| [80-100[ | 4 |
| [100-120[ | 4 |
| [120-140[ | 6 |
| [140-160[ | 2 |
| [160-240[ | 0 |
| [240-260[ | 2 |
| NR | 36 |

**Table S7** – Operating mode and respective number of occurrences (Figure 3f data).

| **Operating mode** | **Number of occurrences (counts)** |
| --- | --- |
| Continuous | 56 |
| Pulsed | 38 |
| NR | 27 |

**Table S8** – Pulse frequency and duty cycle and respective number of occurrences (Figure 3g data).

| **Operating mode** | **Number of occurrences (counts)** |
| --- | --- |
| 5 Hz (50% DC) | 1 |
| 10 Hz | 6 |
| 10 Hz (20-50% DC) | 11 |
| 12.21 Hz | 1 |
| 20 Hz (50% DC) | 1 |
| 40 Hz | 2 |
| 40 Hz (50% DC) | 4 |
| 50 Hz | 2 |
| 73 Hz (35% DC) | 1 |
| 75 Hz (20% DC) | 1 |
| 100 Hz (20% DC) | 1 |
| 1175 Hz (35% DC) | 1 |
| 587 Hz (35% DC) | 1 |
| 3000 Hz | 1 |
| 6000 Hz | 1 |
| 146 000 Hz | 1 |
| Superpulsed | 2 |
